# A Novel Oral GyrB/ParE Dual Binding Inhibitor Effective against Multidrug-Resistant *Neisseria gonorrhoeae* and Other High-Threat Pathogens

**DOI:** 10.1128/aac.00414-22

**Published:** 2022-08-16

**Authors:** Steven Park, Riccardo Russo, Landon Westfall, Riju Shrestha, Matthew Zimmerman, Veronique Dartois, Natalia Kurepina, Barry Kreiswirth, Eric Singleton, Shao-Gang Li, Nisha Mittal, Yong-Mo Ahn, Joseph Bilotta, Kristie L. Connolly, Ann E. Jerse, Joel S. Freundlich, David S. Perlin

**Affiliations:** a Center for Discovery and Innovation, Hackensack Meridian Health, Nutley, New Jersey, USA; b Division of Infectious Disease, Department of Medicine, and the Ruy V. Lourenco Center for the Study of Emerging and Reemerging Pathogens, New Jersey Medical School, Rutgers, The State University of New Jersey, Newark, New Jersey, USA; c Southern Research, Infectious Disease Research, Birmingham, Alabama, USA; d Department of Microbiology and Immunology, Uniformed Services University of the Health Sciences, Bethesda, Maryland, USA; e Department of Pharmacology and Physiology, Rutgers University-New Jersey Medical School, Newark, New Jersey, USA

**Keywords:** *C. difficile*, gyrase, Neisseria, *Neisseria gonorrhoeae*, VRSA, topoisomerase

## Abstract

Drug-resistant Neisseria gonorrhoeae is a serious global health concern. New drugs are needed that can overcome existing drug resistance and limit the development of new resistances. Here, we describe the small molecule tricyclic pyrimidoindole JSF-2414 [8-(6-fluoro-8-(methylamino)-2-((2-methylpyrimidin-5-yl)oxy)-9H-pyrimido[4,5-b]indol-4-yl)-2-oxa-8-azaspiro[4.5]decan-3-yl)methanol], which was developed to target both ATP-binding regions of DNA gyrase (GyrB) and topoisomerase (ParE). JSF-2414 displays potent activity against N. gonorrhoeae, including drug-resistant strains. A phosphate pro-drug, JSF-2659, was developed to facilitate oral dosing. In two different animal models of Neisseria gonorrhoeae vaginal infection, JSF-2659 was highly efficacious in reducing microbial burdens to the limit of detection. The parent molecule also showed potent *in vitro* activity against high-threat Gram-positive organisms, and JSF-2659 was shown in a deep tissue model of vancomycin-resistant Staphylococcus aureus (VRSA) and a model of Clostridioides difficile-induced colitis to be highly efficacious and protective. JSF-2659 is a novel preclinical drug candidate against high-threat multidrug resistant organisms with low potential to develop new resistance.

## INTRODUCTION

Sexually transmitted infections due to Neisseria gonorrhoeae remain a significant global public health concern. Gonorrhea complications affect both women and men, and in women include pelvic inflammatory disease, ectopic pregnancy, and infertility, as well as increased transmission and acquisition of HIV (1). In 2012, the World Health Organization (WHO) estimated that there were 78 million cases among adults worldwide (https://www.paho.org/en/topics/gonorrhea). In 2018, the U.S Centers for Disease Control and Prevention reported a total of 583,405 gonorrhea cases with a national infection rate of 179.1 cases per 100,000 people, which reflects an increase of 63% since 2014 and the highest number since 1991 (2). For decades, gonorrhea was treated successfully using antimicrobials. However, there is now a high prevalence of N. gonorrhoeae strains that are resistant to common antimicrobial classes used for treatment, including sulfonamides, penicillins, cephalosporins, tetracyclines, macrolides, and fluoroquinolones (3). Therapeutic failures with the extended-spectrum cephalosporins, such as cefixime and ceftriaxone, have created a major health crisis. In many countries, ceftriaxone is the only remaining empirical monotherapy for gonorrhea (4, 5). Given the high burden of gonococcal disease with resistance and the rapid emergence of resistant strains to monotherapy, antimicrobial therapy involving high-dose ceftriaxone is recommended (6). However, as anticipated, resistance to this regimen has also occurred (7). It is recognized that N. gonorrhoeae has evolved as a multidrug-resistant superbug representing a major global public health concern (7, 8). The WHO has proposed a 90% reduction in gonorrhea globally (9), although achieving this goal will require overcoming the issue of antimicrobial resistance.

In recent years, new gonorrhea treatment regimens and drug candidates have been introduced to overcome and prevent resistance (4). The most promising class of new drug candidates interferes with DNA biosynthesis by inhibiting bacterial DNA gyrase (GyrB) and topoisomerase IV (ParE) via a unique mechanism (4). DNA gyrase and topoisomerase IV are closely related DNA topoisomerase type II enzymes that are essential for DNA synthesis. These enzymes function in tandem to catalyze topological changes in DNA during replication through supercoil unwinding and subsequent introduction of transient double-stranded DNA breaks and religation and serve as the target for fluoroquinolone therapeutics (10). This dual targeting paradigm confers high susceptibility of new drug candidates against both fluoroquinolone-susceptible and resistant isolates, and furthermore carries a very low probability for development of new resistance.

Zoliflodacin, a first-in-class spiropyrimidinetrione (11, 12), and Gepotidacin, a triazaacenaphthylene inhibitor (13) leverage this dual-targeting approach and are novel, clinical-stage drug candidates that are completing phase 3 trials for the treatment of uncomplicated gonorrhea. These compounds inhibit bacterial DNA gyrase and topoisomerase IV by a novel mode of action that involves a binding site close to, but distinct from that of quinolones. Given their mechanism of action, these agents are broadly active against Gram-negative and Gram-positive bacteria including other biothreat agents such as Streptococcus pneumoniae, Haemophilus influenzae, Clostridium perfringens, and various *Shigella* species (12–15).

The tricyclic pyrimidoindoles are a new class of highly potent molecules that inhibit both GyrB and ParE (TriBE inhibitors) by binding at the highly conserved ATP-binding domain (16). Because the ATP-binding region is separate and distant from the fluoroquinolone-binding domain, it would not be subject to common resistance-associated target site mutations. TriBE inhibitors demonstrate potent broad-spectrum Gram-negative and Gram-positive *in vitro* activity, including against drug-resistant strains, and demonstrated a low mutation frequency of <1.9 × 10^−11^ at 4× the MIC in Escherichia coli (16).

In this report, we describe the potent *in vitro* and *in vivo* properties of the tricyclic pyrimidoindole JSF-2414 and its oral pro-drug conjugate JSF-2659 against drug-sensitive and drug-resistant strains of N. gonorrhoeae, and against drug-resistant Gram-positive pathogens including methicillin- and vancomycin-resistant Staphylococcus aureus (MRSA and VRSA) and Clostridioides difficile.

## RESULTS

### Chemical and pharmacokinetic properties of JSF-2414 and JSF-2659.

**FIG 1:**
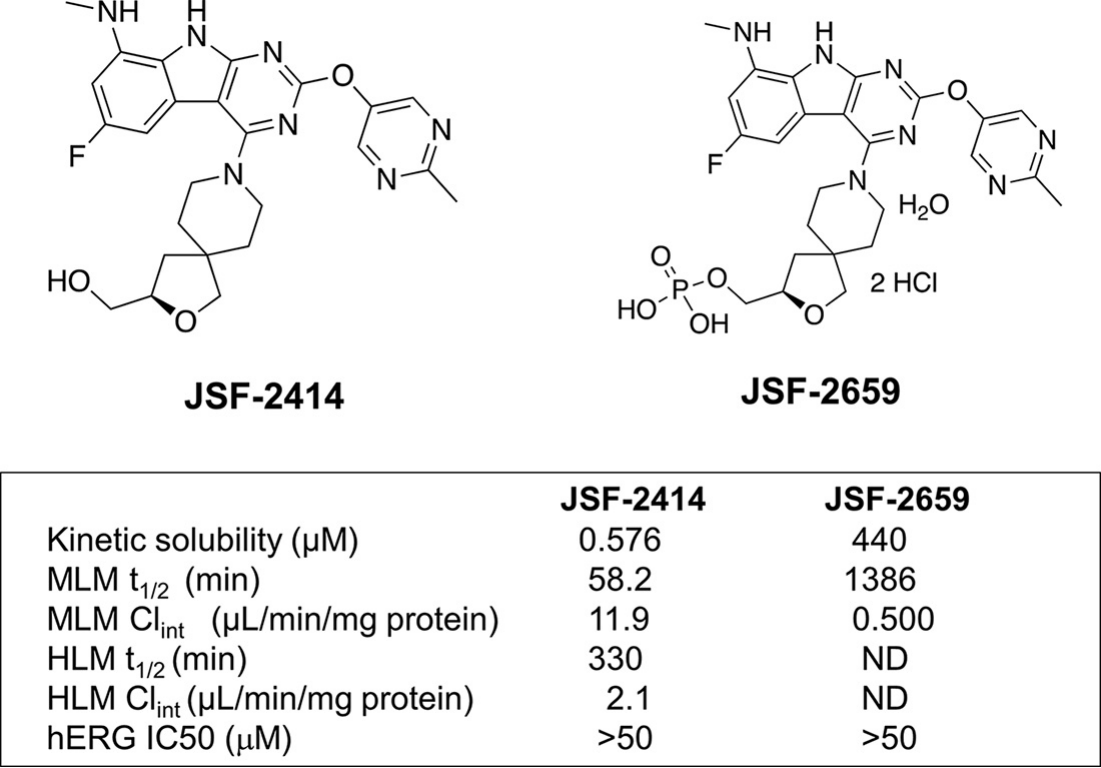
Chemical structures for JSF-2414 (8-(6-fluoro-8-(methylamino)-2-((2-methylpyrimidin-5-yl)oxy)-9H-pyrimido[4,5-b]indol-4-yl)-2-oxa-8-azaspiro[4.5]decan-3-yl)methanol and phosphate derivative JSF-2659 showing kinetic aqueous solubility and metabolic stability, including half-life (t_1/2_) and intrinsic clearance (Cl_int_) parameters with isolated mouse liver microsomes (MLM) and human liver microsomes (HLM).

The small molecule compound 8-(6-fluoro-8-(methylamino)-2-((2-methylpyrimidin-5-yl)oxy)-9H-pyrimido[4,5-b]indol-4-yl)-2-oxa-8-azaspiro[4.5]decan-3-yl)methanol (JSF-2414) (Fig. 1) was selected from a series of tricyclic pyrimidoindoles with potent activity (MIC < 0.05 μg/mL) against the fastidious Gram-negative organism N. gonorrhoeae and the Gram-positive organism S. aureus. JSF-2414 has a molecular weight of 493.54 g/mol. Its solubility, metabolic stability, hERG inhibition profile, and *in vitro* intrinsic clearance (CL_int_) in mouse and human liver microsomes are summarized in Fig. 1. The *in vitro* metabolic stability of the compound in the presence of mouse or human liver microsomes was in an acceptable dosing range (i.e., half-life [t_1/2_] ≥ 60 min) (17). The hERG 50% inhibitory concentration (IC_50_) value of JSF-2414 was >50 μM, demonstrating a very low blocking potential of cardiac channels. The relatively low kinetic solubility of JSF-2414 (0.576 μM) was addressed by synthesizing a phosphate prodrug candidate, JSF-2659 (Fig. 1, synthetic scheme in Supplemental Methods), which increased the solubility of the parent drug candidate 764-fold to 440 μM. The pharmacokinetics (PK) of JSF-2659 administered orally at 25 mg/kg showed a t_1/2_ of 2.03 ± 0.25 h and a maximum concentration in the serum (*C*_max_) of ~3,500 ng/mL of JSF-2414, while intramuscular (IM) administration of JSF-2659 at 25 mg/kg increased the t_1/2_ to 12.5 ± 0.25 h while the *C*_max_ decreased slightly to ~2,400 ng/mL.

### Antimicrobial activity against *Neisseria gonorrhoeae* and other high-threat drug-resistant pathogens.

The antimicrobial properties of JSF-2414 (parent drug candidate) were evaluated by the Southern Research Institute against a collection of 96 N. gonorrhoeae clinical isolates, including drug-resistant strains provided by the CDC under contract with the National Institutes of Health (see Methods). An agar dilution method was used according to guidelines established by the Clinical and Laboratory Standards Institute (CLSI), along with six control antibacterial agents (azithromycin, cefixime, ceftriaxone, ciprofloxacin, penicillin, and tetracycline) which served as reference controls. JSF-2414 was highly active against all drug-susceptible and drug-resistant strains, with an MIC_90_ of 0.0078 μg/mL (range: 0.0005 to 0.03 μg/mL) (Table 1). Individual MIC values for each strain are listed in Supplemental Table S1.

**TABLE 1:** MIC summary of N. gonorrhoeae and clinically important Gram-positive bacteria^*a*^

Species	Test and control articles								
	JSF-2414	Azi	Cfx	Cro	Cip	Pen	Tet	Van	Rif
N. gonorrhoeae									
*N*	96	96	96	96	96	96	96		
MIC_50_	0.0039	0.125	0.125	0.031	8	1	1		
MIC_90_	0.0078	2	0.25	0.063	16	2	1		
MIC range	0.0005–0.03	0.016–>16.0	0.016–4	<0.002–0.125	<0.016–16	<0.008–4	0.063–16		
MRSA									
*N*	101				101			8	
MIC_50_	0.031				>4			NA	
MIC_90_	0.062				>4			NA	
MIC range	0.002–0.125				0.031–>4			0.5–1	
VRSA									
*N*	7							9	7
MIC_50_	NA							NA	N/A
MIC_90_	NA							NA	N/A
MIC range	0.0156–0.031							<1.56–>200	<0.078–>10
VISA									
*N*	8							8	8
MIC_50_	NA							NA	N/A
MIC_90_	NA							NA	N/A
MIC range	0.0156–0.063							3.125–12.5	<0.078–>10
VRE									
*N*	8				8			8	
MIC_50_	NA				NA			NA	
MIC_90_	NA				NA			NA	
MIC range	0.002–0.031				^*b*^			0.25–>4	
MRSE									
*N*	4				4			4	
MIC_50_	NA				NA			NA	
MIC_90_	NA				NA			NA	
MIC range	0.002–0.004				0.062–0.5			0.5–1	
C. difficile									
*N*	9							9	
MIC_50_	NA							NA	
MIC_90_	NA							NA	
MIC range	0.0078–0.0630							0.5–1	
B. anthracis									
*N*	2				2				
MIC_50_	NA				NA				
MIC_90_	NA				NA				
MIC range	0.049–0.098				0.031–0.125				

a Azi, azithromycin; Cfx, cefixime; Cro, ceftriaxone; Cip, ciprofloxacin; Pen, penicillin; Rif, rifampicin; Tet, tetracycline; Van, vancomyin; MRSA, methicillin-resistant Staphylococcus aureus; MRSE, methicillin-resistant Staphlyococcus epidermidis; VISA, vancomycin-intermediate Staphylococcus aureus; VRSA, vancomycin-resistant S. aureus; VRE, vancomycin-resistant enterococci; NA, not applicable. All MIC values (MIC_50_ and MIC_90_) are given in μg·mL^−1^. MIC value ranges are reported for strains with a small population (*n* < 10).

b VRE strains had the same MIC value of >4 for ciprofloxacin.

Further *in vitro* susceptibility testing was performed against a highly diverse panel of Gram-positive and Gram-negative bacterial species. JSF-2414 showed prominent growth inhibition against 101 clinical MRSA strains, with a modal MIC of 0.031 μg/mL (range: 0.002 to 0.125 μg/mL). Because JSF-2414 binds at a distance from the region of gyrase that confers resistance to fluoroquinolones, all ciprofloxacin-resistant strains with an MIC of >4 μg/mL were fully sensitive (modal MIC = 0.062 μg/mL; range: 0.002 to 0.125 μg/mL; *n* = 30). JSF-2414 also showed potent activity against vancomycin-intermediate *Staphylococcus aureus* (VISA) (MIC range: 0.0156 to 0.156; *n* = 8) and VRSA (MIC range: 0.0156 to 0.063; *n* = 7) strains (Table 1).

JSF-2414 was also highly active against Staphylococcus epidermidis (MIC range: 0.002 to 0.004 μg/mL; *n* = 4), Enterococcus faecium (MIC range: 0.004 to 0.008; *n* = 3), vancomycin-resistant enterococci (VRE) (MIC range: 0.002 to >0.5 μg/mL; *n* = 8), E. faecalis (MIC range: 0.002 to 0.008; *n* = 3), and B. anthracis (MIC range: 0.049 to 0.098 μg/mL; *n* = 2) (Table 1). JSF-2414 was assessed for activity against a toxigenic C. difficile panel that included the common ribotypes 001, 002, 012, 014, 020, 038, 078, and 087 as well as the highly virulent ribotype 027. All C. difficile strains tested were highly susceptible to JSF-2414, with MICs ranging from 0.0078 to 0.063 μg/mL (Table 1). In general, there was weak activity (MIC > 0.5 μg/mL) against the Gram-negative pathogens E. coli, Pseudomonas aeruginosa, Klebsiella pneumoniae, Acinetobacter baumannii, and select agents Francisella tularensis and Yersinia pestis (data not shown). In MIC studies with N. gonorrhoeae and S. aureus, spontaneous resistance was not observed with frequencies of <7.5 × 10^−4^ at 1× MIC for Neisseria and <5.0 × 10^−9^ at 1× MIC for MRSA (data not shown).

### Efficacy of JSF-2659 in *Neisseria gonorrhoeae* vaginal colonization models.

**FIG 2:**
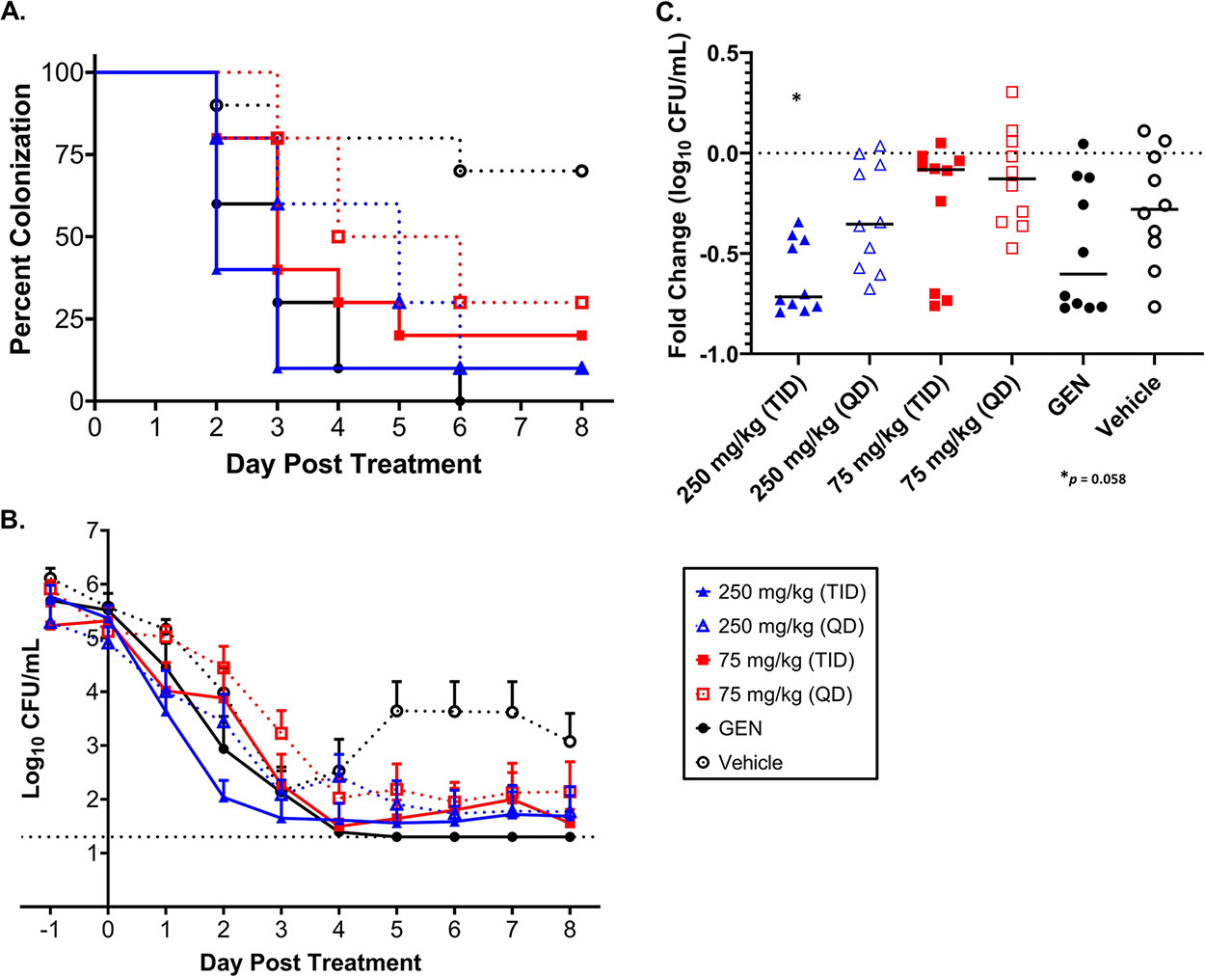
Dose-dependent efficacy of JSF-2659 against the multidrug-resistant Neisseria gonorrhoeae strain H041 in the murine vaginal infection model. Animals were vaginally inoculated with H041 bacteria and 2 days later, treated daily with JSF-2659 as shown in Supplemental Fig. S1. Mice received daily oral doses of JSF-2659 at 75 mg/kg QD (quaque die or once daily) (red outlined square), 75 mg/kg TID (ter in die, 3 × a day) (red solid square), 250 mg/kg QD (blue outlined triangle), or 250 mg/kg TID (blue solid triangle) over 8 days. Gentamicin at 48 mg/kg, 5 QD (black solid circle) was used as a positive control; the vehicle was used as the negative-control (black outlined circle). (A) A dose-dependent clearance rate was observed in JS-2659-treated mice as assessed by the percentage of culture-positive mice on eight consecutive days following treatment initiation. *P* values of 0.02, 0.01, 0.003, and 0.0007 for the 75 mg/kg TID, 250 mg/kg QD, 250 mg/kg TID, and gentamicin control group versus the vehicle control group, respectively (log-rank Mantel-Cox test). (B) Average number of CFU recovered per mL of vaginal swab suspension declined over time in all antibiotic treatment groups compared to that in the vehicle control group, with the differences between the 250 mg/kg TID group and the gentamicin control groups being significantly different than the vehicle control group (*P* = 0.004 and 0.007, respectively; 2-way analysis of variance [ANOVA] with repeated measures, Bonferroni *post hoc* analysis). (C) Fold change in CFU/mL between day 0 (pre-treatment) and 48 h post-treatment showed a trend toward a significant reduction for the 250 mg/kg TID treatment group (*P* = 0.058).

JSF-2659 is rapidly and completely (>99%) converted by host phosphatases to its highly active form JSF-2414 following oral administration in mice (data not shown). The *in vivo* efficacy of JSF-2659 was initially assessed in a well-characterized estradiol-treated mouse model of cervico-vaginal infection at the Uniformed Services University (USU) (18) against the multidrug-resistant strain H041 (JSF-2414 MIC = 0.0078 μg/mL) (19), which carries resistance to extended-spectrum cephalosporins, tetracycline, macrolides, and several fluoroquinolones, including ciprofloxacin (18). The detailed experimental design of the model is shown in Supplemental Figure S1. JSF-2659 administered at doses of 75 mg/kg TID (ter in die, 3 × a day) (q: 6 h), 250 mg/kg QD quaque die or (once daily) or 250 mg/kg TID (q: 6 h) showed a significant reduction in the percentage of mice colonized with strain H041 over 8 culture days after treatment (Fig. 2A), as well as a reduction in vaginal tract burdens (CFU/mL recovered) (Fig. 2B). However, after 48 h of treatment, the average CFU/mL recovered was significantly reduced compared to the vehicle control only after 250 mg/kg TID treatment (*P = *0.058) (Fig. 2C). After day 8 post-treatment, significant reductions in bacterial burden were observed between the three JSF-2659 treatment groups and the vehicle control (Fig. 2B). All JSF-2659 treatment groups had comparable reductions in bacterial burden relative to the gentamicin (GEN)-treated control. It was observed that microbial burdens in some animals were elevated following treatment (Supplemental Fig. S2). However, in follow-up studies, there was no indication of resistance development or change in susceptibility (not shown).

**FIG 3:**
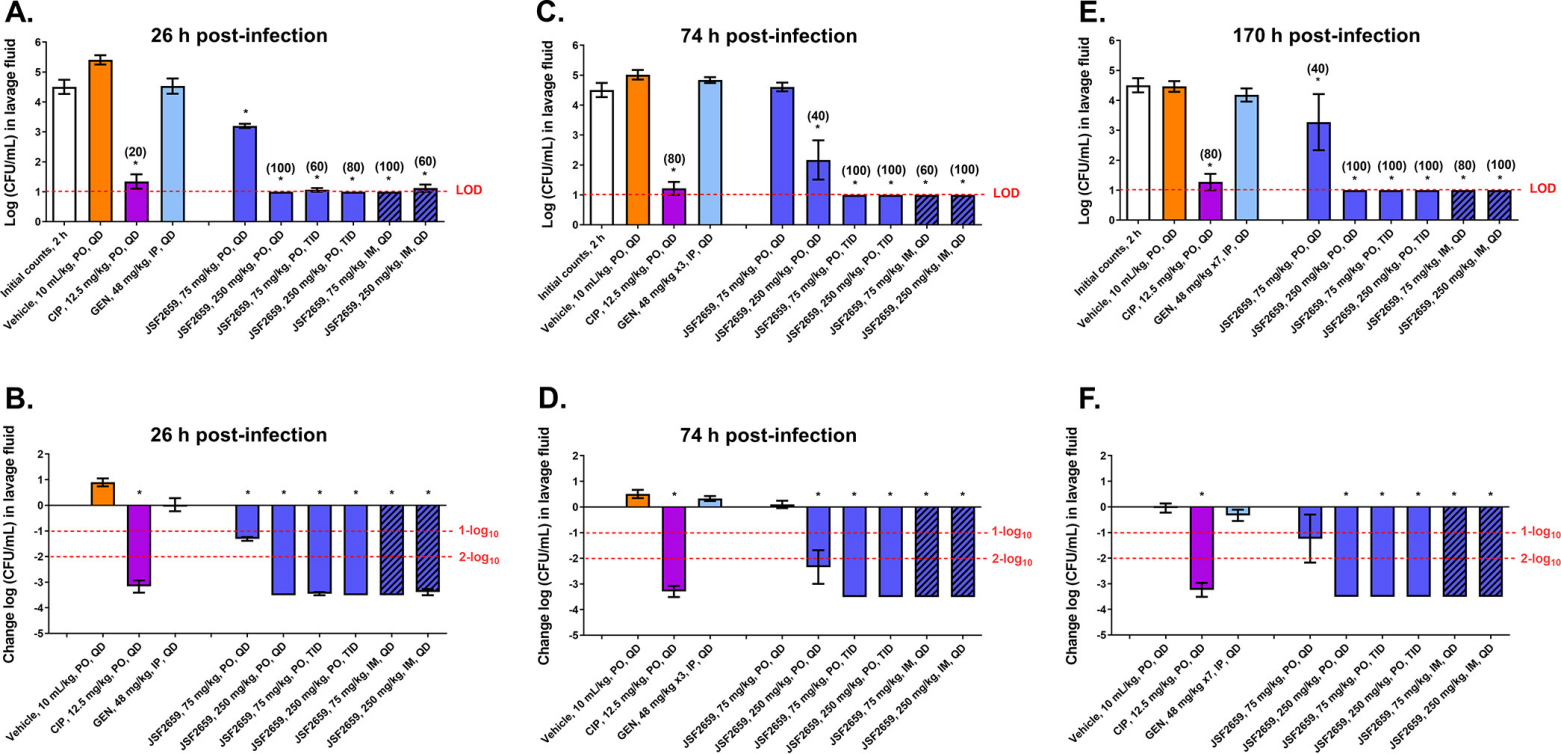
Effects of JSF-2659, ciprofloxacin, and gentamicin in a N. gonorrhoeae (ATCC 700825) intravaginal infection model using ovariectomized BALB/c mice. Total bacterial counts (A, C, and E) and a net change in counts (B, D, and F) is shown for vaginal lavage fluids following test article treatment relative to the initial 2-h counts at the time of dosing. JSF-2659 at 75 and 250 mg/kg was administered orally (PO) with two dosing schedules, including once at 2 h after infection and three times (TID) with a 6-h interval at 2, 8, and 14 h after infection. In addition, JSF-2659 at 75 and 250 mg/kg was administered intramuscularly (IM) once (QD) at 2 h after infection. Two reference compounds were applied in this study: ciprofloxacin (CIP) and gentamicin (GEN). Ciprofloxacin was administered orally at 12.5 mg/kg once at 2 h after infection. Gentamicin was administered intraperitoneally (IP) at 48 mg/kg once at 2 h after infection for seven consecutive days. Animals were sacrificed at 26 h (A and B), 72 (C and D), and 170 h (E and F) after infection. Vaginal lavage was performed, and the bacterial suspensions were plated onto chocolate agar to determine the N. gonorrhoeae counts. Asterisks (*) indicate significant differences (*P* < 0.05) compared to the respective vehicle control as determined by one-way ANOVA followed by Dunnett’s test. Percentage clearance in parentheses represents the percentage of animals with counts below the limit of detection (LOD).

The promising efficacy of JSF-2659 was independently assessed via PO and intramuscular administration in a different Neisseria gonorrhoeae vaginal colonization model using the antibiotic-susceptible strain ATCC 700825 (JSF-2414 MIC = 0.0039 μg/mL). In this model, ovariectomized female BALB/c mice were used and they were given subcutaneous (SC) injections of 17β-estradiol. The influence of vaginal bacteria was minimized by treating animals twice daily (BID) 2 days prior to infection with streptomycin and vancomycin, along with trimethoprim sulfate at 0.4 mg/mL (see Materials and Methods). Oral administration (PO) dosing and an alternative IM treatment route were tested with JSF-2659 at 75 and 250 mg/kg QD at 2 h after vaginal inoculation with bacteria. A single-dose IM route was evaluated because of the long PK (t_1/2_ = 12.5 h) observed for JSF-2659 following IM administration. At 26 h postinfection and 2 h post-therapy, a significant dose-dependent reduction in the bacterial counts was observed, resulting in a 3.51-log_10_ killing effect to the limit of detection (LOD) following PO administrations of JSF-2659 at 75 and 250 mg/kg TID and JSF-2659 at 250 mg/kg QD, as well as 3.51- and 3.39-log_10_ killing effects following the IM administrations of JSF-2659 at 75 and 250 mg/kg QD, respectively, relative to control (*P < *0.05). A 1-log_10_ killing effect was observed with the PO administration of JSF-2659 at 75 mg/kg QD relative to the baseline group (*P < *0.05). A 3.17-log_10_ killing effect was observed with the PO administration of reference ciprofloxacin (CIP) at 12.5 mg/kg QD relative to the baseline group (*P < *0.05). No significant reduction in the bacterial counts was observed with the IP administration of GEN at 48 mg/kg QD relative to the vehicle control group or the baseline group at 26 h (Fig. 3A and B). Similar reductions in bacterial burdens were observed at 72 h (Fig. 3C and D) and 170 h (Fig. 3E and F) following initiation of treatment. In summary, >3-log_10_ killing effects relative to the baseline group were observed in animals euthanized at 26, 76, and 170 h after infection with (i) PO administrations of JSF-2659 at 75 and 250 mg/kg TID; (ii) PO administrations of JSF-2659 at 250 mg/kg QD; (iii) IM administrations of JSF2659 at 75 and 250 mg/kg QD; and (iv) PO administration of reference CIP at 12.5 mg/kg QD.

### Efficacy of JSF-2659 against VRSA in a deep soft tissue infection model.

**FIG 4:**
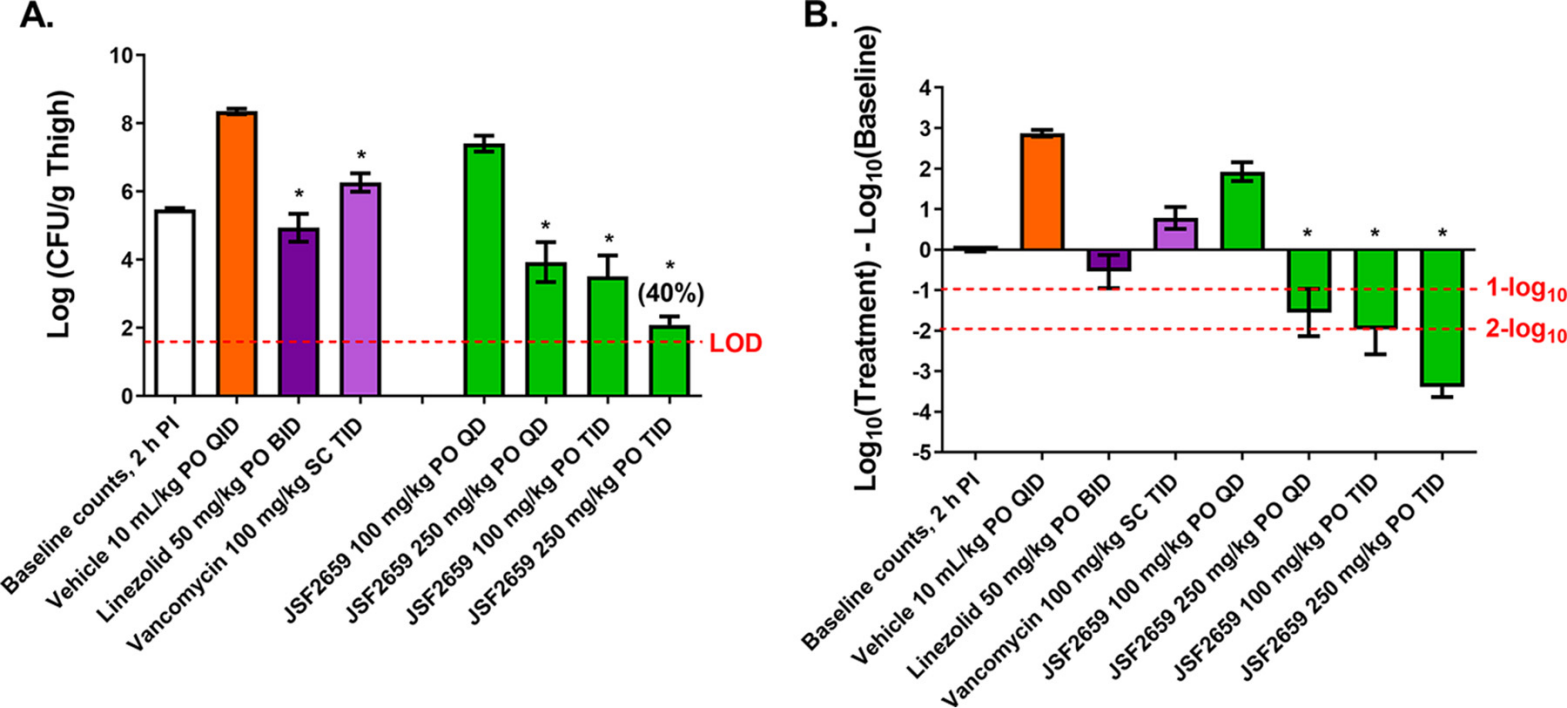
Effects of JSF-2659, linezolid, and vancomycin in the S. aureus (vancomycin-resistant S. aureus [VRSA], VRS-2) thigh infection model with neutropenic mice. Mice were induced neutropenic with cyclophosphamide treatment and were then inoculated intramuscularly with an inoculation density of 1.0 × 10^5^ CFU/mouse. JSF-2659 at 100 and 250 mg/kg was administered orally (PO) with two dosing schedules, including once at 2 h after infection and three times with a 6-h interval at 2, 8, and 14 h after infection. Two reference compounds were applied in this study: linezolid and vancomycin. Linezolid was administered orally at 50 mg/kg twice at 2 and 14 h after infection. Vancomycin was administered subcutaneously (SC) at 100 mg/kg three times at 2, 8, and 14 h after infection. Animals were euthanized at 2 or 26 h, the thigh tissues were harvested, and the total bacterial counts (CFU/g) (A) and changes in counts (B) in thigh tissues were compared. Asterisks (*) indicate significant differences (*P* < 0.05) compared to the vehicle control as determined by one-way ANOVA followed by Dunnett’s test. Linezolid, administered orally at 50 mg/kg twice at 2 and 14 h after infection, and vancomycin, administered subcutaneously (SC) at 100 mg/kg TID at 2, 8, and 14 h after infection, served as reference controls. One infected but untreated group was euthanized at 2 h after infection for the initial bacterial counts. Mice from the test article, reference compound, and vehicle control treatment groups were euthanized at 26 h after infection. The thigh tissue was excised for bacterial enumeration (CFU/gram). A one-way ANOVA followed by Dunnett’s comparison test was performed to assess statistical significance (*P* < 0.05) for the bacterial counts of the treated animals compared to those of the vehicle control group. Percentage clearance in parentheses represents the percentage of animals with counts below the LOD.

JSF-2414 displayed potent *in vitro* activity against strains of MRSA, VRSA, and VISA (Table 1). The oral efficacy of JSF-2659 against VRSA was assessed in a neutropenic murine thigh infection model. Female ICR mice were rendered neutropenic with cyclophosphamide treatment and then inoculated intramuscularly with VRSA (VRS-2, JSF-2414 MIC = 0.031 μg/mL) at 1.03 × 10^5^ CFU/mouse. JSF-2659 at 100 and 250 mg/kg was administered orally with two dosing schedules, including QD 2 h after infection and TID at 6-h intervals 2, 8, and 14 h after infection. Linezolid and vancomycin served as reference standards. JSF-2659 PO administrations at 250 mg/kg QD and 100 and 250 mg/kg, resulted in significant bacterial count reductions of 0.95 and 4.42 log_10_, respectively, relative to the vehicle control group (Fig. 4). When administered PO TID at 100 and 250 mg/kg, JSF-2659 yielded further bacterial burden reductions of 4.84 and 6.26 log_10_, respectively (Fig. 4B). PO and SC administration of the respective reference agents, linezolid at 50 mg/kg BID and vancomycin at 100 mg/kg TID, yielded a reduction in bacterial counts of 3.41 and 2.09 log_10_, respectively.

### Efficacy of JSF-2659 against *C. difficile* in a colitis model.

**FIG 5:**
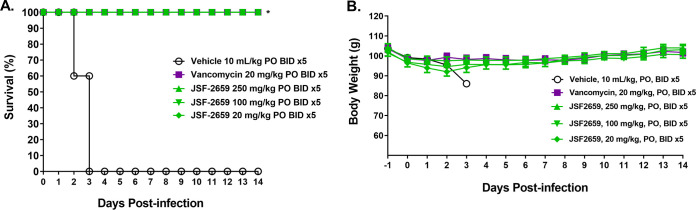
Kaplan-Meier plot of 14-day survival in the C. difficile ATCC BAA-1805 hamster colitis model. Animals were inoculated orally (PO) with C. difficile ATCC BAA-1805 at 5.8 × 10^5^ spores/animal. Test article JSF2659 at 20, 100, and 250 mg/kg, and reference agent, vancomycin at 20 mg/kg, were administered orally twice daily (BID) with an 8-h interval starting at 16 h after infection for five consecutive days. Animal mortality was monitored for 14 days (A). Asterisk (*) indicates a significant increase (*P* < 0.05) in the survival rates of the treated animals compared to the vehicle control group at the 14-day time point as determined by Fisher’s exact test. The test article, JSF2659 at 20, 100, and 250 mg/kg, and the reference agent, vancomycin at 20 mg/kg (points submerged behind JSF2659 plots), all showed significance and are indicated. (B) Body weight changes of C. difficile ATCC BAA-1805-infected hamsters. Animals were inoculated orally with C. difficile ATCC BAA-1805 at 5.8 × 10^5^ spores/animal. The test article, JSF2659 at 20, 100, and 250 mg/kg, and the reference agent, vancomycin at 20 mg/kg, were administered orally twice daily with an 8-h interval starting at 16 h after infection for five consecutive days. Body weight changes were monitored for 14 days.

The protective efficacy of JSF-2659 in a hamster C. difficile colitis model was evaluated. The study was performed with a lethal dose (LD_90–100_) of strain C. difficile BAA-1805 (ribotype 027/NAP1/BI; JSF-2414 MIC = 0.031 μg/mL). Test animals were pretreated with a single SC administration of clindamycin at 10 mg/kg on day −1 to render the animals vulnerable to C. difficile infection. On day 0, animals were inoculated orally with C. difficile BAA-1805 at 5.8 × 10^5^ spores/animal. JSF-2659 at 20, 100, and 250 mg/kg and the reference agent, vancomycin at 20 mg/kg, were administered PO twice daily with an 8-h interval, starting at 16 h after infection, for five consecutive days. Animal mortality and body weight changes were monitored for 14 days. Infection with C. difficile resulted in 100% mortality during the 14-day observation period (Fig. 5A). All test animals survived the complete study period after treatment with JSF-2659 at 20, 100, and 250 mg/kg PO BID for 5 days (100% survival for all groups, *P* < 0.05) (Fig. 5A). The PO administration of vancomycin at 20 mg/kg resulted in 100% survival over the treatment period (*P < *0.05). No significant loss of body weight for the test animals relative to the positive controls was observed during the 14-day observation period, consistent with compound efficacy (Fig. 5B).

## DISCUSSION

Despite the high worldwide burden of gonorrhea infections, the number of effective treatment options for gonorrhea infections is limited and further diminished by the emergence of multidrug resistance. This has prompted the WHO to place N. gonorrhoeae on a global priority list of antibiotic-resistant pathogens for which there is an urgent need to develop novel antimicrobial therapeutics (20). In this report, we describe the potent *in vitro* and *in vivo* antimicrobial properties of JSF-2414, a novel tricyclic pyrimidoindole which, based on previous studies with other TriBE inhibitors, binds to the ATP-binding domains of both DNA gyrase (GyrB) and topoisomerase IV (ParE). This tricyclic pyrimidoindole (Fig. 1) emerged from a study identifying a low-molecular-weight fragment scaffold with suitable hydrogen-bond donor/acceptor moieties that could engage the ATP adenine-binding aspartate and structural water in the active site pocket of Enterococcus faecalis GyrB, and it is a racemate of a patented scaffold from the published study (16, 21). Because gyrase and topoisomerase are well-established pharmacologic targets, targeting the ATP-binding site is important since it is separate from the drug-binding site of fluoroquinolones and resistance-mediated mutations within the site (22, 23). It is not surprising that JSF-2414 was highly active (MIC range: 0.0005 to 0.003 μg/mL) against a CDC panel (*n* = 96) of clinical isolates of N. gonorrhoeae displaying a wide variety of drug resistances, including fluoroquinolone resistance (Table 1). This potent level of activity likely reflects the nature of its target and binding properties, which were originally optimized from structure-based studies (16).

The development of drugs targeting the ATP-binding domain is not new, as discovery programs for decades have focused on inhibitors (e.g., benzothiazole, tetrahydrobenzothiazole, etc.) which target the ATP-binding/hydrolysis sites on GyrB and ParE (22–33). Indeed, in the 1950s, the natural product novobiocin was reported as being potently bactericidal to Gram-positive bacteria, and in the 1970s it was demonstrated to inhibit DNA replication (34) that eventually led to the ATP-binding domain of GyrB. It was licensed for clinical use under the tradename Albamycin (Pharmacia and Upjohn) in the 1960s but was withdrawn due to poor efficacy (35). Newer efforts have also been unsuccessful in generating an inhibitor series with broad spectrum antibacterial activity or advancing a molecule into the clinic. Most recently, the tetrahydroquinazoline and 4,5,6,7-tetrahydrobenzo[1,2-d] thiazole scaffolds were originally identified as low micromolar inhibitors of the DNA gyrase ATP-binding domain (27). However, the compounds showed modest antibacterial activity, appeared to be substrates for efflux transporters, and poorly penetrated the cell wall (27). In contrast to these efforts, JSF-2414, the tricyclic pyrimidoindole in this study, was highly active against a broad range of clinical isolates displaying a variety of resistance mechanisms (Supplemental Table S1).

Potent but more modest MICs have also been reported for later-stage clinical candidates which target more classical regions of gyrase. The spiropyrimidinetrione zoliflodacin is presumed to bind to the same pocket in gyrase as fluoroquinolones based on X-ray crystallography structure analysis (12). However, it inhibits gyrase through a separate mechanism (36), which enables it to be effective against most fluroquinolone-resistant strains (12). In a multi-laboratory quality study of zoliflodacin against standardized ATCC strains by eight independent laboratories using CLSI document M23, the agar dilution MIC QC range for zoliflodacin against the N. gonorrhoeae QC strain ATCC 49226 was defined as 0.06 to 0.5 μg/mL (37). In a separate study, zoliflodacin showed potent antibacterial activity against multidrug-resistant strains of N. gonorrhoeae with MICs ranging from ≤0.002 to 0.25 μg/mL (12). The triazaacenaphthylene inhibitor Gepotidacin has been shown to form an antibacterial complex with S. aureus DNA gyrase and DNA, demonstrating a novel mechanism of inhibition that overcomes fluoroquinolone resistance. This inhibitor was demonstrated to bind close to the fluoroquinolone-binding site, where it appears to span DNA and a transient noncatalytic pocket on the GyrA dimer (38). A multi-laboratory quality assurance study demonstrated a modal MIC of 0.5 μg/mL against the N. gonorrhoeae QC strain ATCC 49926 (39).

A hallmark of DNA gyrase inhibitors is their broad-spectrum activity, especially against Gram-positive organisms. JSF-2414 showed potent cross-activity against MRSA with an MIC of 0.031 mg/mL (range 0.002–0.125 μg/mL), including VISA and VRSA strains (Supplemental Table S2), with comparable activity against 30 clinical isolates with fluoroquinolone resistance of MIC > 4 μg/mL. It showed comparable high activity (low MICs) against S. epidermidis, E. faecium, VRE, E. faecalis, B. anthracis, multidrug-resistant tuberculosis, and C. difficile. Gepotidacin showed *in vitro* activity with an MIC_90_ of 0.5 μg/mL and equivalent activity against S. pneumoniae (13). Similarly, zoliflodacin, in studies involving thousands of clinical isolates, has shown MIC values of 0.12 to 0.5 μg/mL for S. aureus ATCC 29213, 0.25 to 2 μg/mL for E. faecalis ATCC 29212, 0.12 to 0.5 μg/mL for S. pneumoniae, and 0.12 to 1 μg/mL for H. influenzae (36, 40). For all of the inhibitors, the activity was less than fluroquinolones against prominent Gram-negative organisms like E. coli and P. aeruginosa. For JSF-2414, gepotidacin, and zoliflodacin, MIC values were 0.5, 4, and 4 μg/mL versus E. coli, respectively (13, 37). However, they did provide consistent MIC activity against fluroquinolone-resistant isolates.

To investigate the *in vivo* potential of JSF-2414 against N. gonorrhoeae infection, a phosphate prodrug candidate JSF-2659 (Fig. 1) was developed to facilitate oral dosing. The *in vivo* efficacy of JSF-2414 was demonstrated in two separate N. gonorrhoeae genital tract infection models. In the first model with MDR strain H041, dosing of JSF-2659 at 75 mg/kg TID (q: 6 h), 250 mg/kg QD, or 250 mg/kg TID (q: 6 h) showed a significant reduction in the percentage of infected mice over 8 culture days after treatment (*P ≤ *0.04 for 75 TID, 250 TID) (Fig. 2). In the second model, dose-dependent oral administration of JSF-2659 profoundly reduced the burden of drug susceptible N. gonorrhoeae (ATCC 700825) by ~4.5 log_10_ to the limit of detection. Again, a PO dose-dependent response was observed (Fig. 3). A single IM administration at 75 or 250 mg/kg was effective in eliminating viable burdens (LOD) (*P < *0.05 compared to vehicle control). These promising preliminary data suggest that high exposures of the drug candidate are critical for the strong pharmacodynamic response. This may imply a *C*_max_ component to this compound, although this will be addressed in a more detailed pharmacokinetic-pharmacodynamic modeling studies. In addition, the high serum protein binding of 98.8% may contribute to the need for higher dosing levels. The potential for a single IM injection to control N. gonorrhoeae is appealing, especially in clinical settings where repeated drug dosing and compliance may not be easy to achieve.

Finally, given the potent inhibitory activity of JSF-2414 against a range of high-threat Gram-positive organisms, a preliminary study was initiated to explore the *in vivo* potential of JSF-2659 against MRSA and C. difficile. A murine deep-tissue thigh model was used to demonstrate a dose-dependent reduction of VRSA and maximum >6.2-log_10_ reduction to near the LOD when administered PO at 250 mg/kg TID (Fig. 4). In a hamster colitis model, JSF-2659 administered PO at 20, 100, or 250 mg/kg BID for 5 days was fully efficacious in preventing C. difficile-induced mortality over 14 days (max) relative to the untreated controls (Fig. 5).

In summary, the preclinical small molecule JSF-2414 is a tricyclic pyrimidoindole which was designed to target the ATP-binding/hydrolysis region of DNA gyrase (GyrB) and topoisomerase (ParE). JSF-2414 displays highly potent activity against the fastidious Gram-negative organism N. gonorrhoeae, including fluoroquinolone-resistant and other drug-resistant strains. In preliminary *in vivo* efficacy studies, oral administration of JSF-2659, a phosphate prodrug candidate, was highly efficacious against N. gonorrhoeae in reducing microbial burdens to the limit of detection in two different animal models of vaginal infection. Lastly, due to its potent activity against Gram-positive organisms *in vitro*, JSF-2659 was shown in a preliminary preclinical deep tissue model of MRSA and model of C. difficile-induced colitis to be highly efficacious and protective. This promising new preclinical development candidate has strong potential to be used against high-threat multidrug-resistant organisms. Its mechanism of action as a dual ATP-binder ensures that the development of resistance will be extremely low.

## MATERIALS AND METHODS

### Bacterial strains.

N. gonorrhoeae clinical isolates (*n* = 96) were obtained as frozen stocks from the Centers for Disease Control and Prevention (41). The strains were inventoried using the original designations and stored in a freezer set at −70°C; simple ID numbers (CDC1 to CDC100) were assigned for internal use. The isolates were plated onto Chocolate II Agar (BD BBL no. 221267) to assess purity and growth on solid medium at 36 ± 1°C in an atmosphere of 5% CO_2_. Plates were visually inspected for colony growth and morphology after 24 h incubation. All isolates formed smooth, nonpigmented, small colonies characteristic of N. gonorrhoeae. The Gram-positive bacteria used in this study included clinical isolates of S. epidermidis, MRSA, VRSA, E. faecium, VRE, and E. faecalis, obtained from the Kreiswirth Laboratory at the Hackensack Meridian Health Center for Discovery and Innovation (HMH-CDI). B. anthracis was obtained from BEI Resources, and a toxigenic C. difficile panel that included the common ribotypes 001, 002, 012, 014, 020, 038, 078, 087, and the highly virulent ribotype 027 was obtained through PDS/Eurofins. Gram-negative pathogens included E. coli, P, aeruginosa, K. pneumoniae and A. baumannii (Kreiswirth Laboratory, HMH-CDI), and the select agents F. tularensis *and* Y. pestis (BEI Resources).

### Susceptibility testing.

**(i) Neisseria gonorrhoeae.** Susceptibility testing was performed by the Southern Research Institute (Birmingham, AL) under NIH testing contract no. HHSN272201100012I. Six control antibacterials, azithromycin (Azi), cefixime (Cfx), ceftriaxone (Cro), ciprofloxacin (Cip), penicillin (Pen), and tetracycline (Tet), were purchased from commercial sources. Hundred-fold concentrated stock solutions of test compounds and control antibacterials were prepared in the appropriate solvent. Dimethyl sulfoxide (DMSO) was used for the test compound and for Azi, Cfx, Pen, and Tet. The Cro stock solution was made with sterile water and the Cip stock was made with 0.1 N HCl_(aq)_. Potency values provided by the manufacturers of the drugs were used to calculate the amounts of powder needed to prepare stock solutions with a final concentration of 3.2 mg·mL^−1^ (Cip), 1.6 mg·mL^−1^ (Azi, Pen, Tet), and 0.4 mg·mL^−1^ (Cfx, Cro). The potency of the test compound powders was assumed to be 100% for the purpose of this study. The stock solutions of 50 μg·mL^−1^ compound were prepared in DMSO. The stock solutions were used as starting material for 2-fold serial dilutions in corresponding solvents to obtain compound solutions that could be directly added at a ratio of 1:100 (vol/vol %) to molten Gonococci agar (GC agar) before pouring rectangular assay plates with the same dimensions as the standard 96-well microplates (Thermo Scientific no. 267060). The GC agar recommended for N. gonorrhoeae testing contains 3.6% GC agar base (Oxoid GC agar no. CM0367) and 1% defined growth supplement (BD BBL IsoVitaleX no. 211876). The compound solutions described above were incorporated into GC agar medium to pour rectangular plates which represented 2-fold serial dilutions consisting of 12 compound dilution steps of test or control article. The serial dilutions in agar medium were prepared in triplicate. JSF-2414 was tested in the range between 0.500 and 0.00024 μg·mL^−1^. For Azi, Pen, and Tet, the test range was 16.00 to 0.008 μg·mL^−1^; Cfx and Cro were tested in a range of 4.00 to 0.002 μg·mL^−1^; and Cip was assayed in a range of 32.00 to 0.016 μg·mL^−1^. Strains were grown on drug-free agar plates (BD BBL Chocolate II Agar plates) for 24 h at 36 ± 1°C in 5% CO_2_. Colonies were suspended directly in 0.9% saline supplemented with 0.5× Mueller-Hinton medium. In preparation for the MIC assay, the inocula were standardized by adjusting their turbidity to 0.5 McFarland standard and transferred by dispensing 700-μL aliquots to 96-well deep well plates. The deep well plates with the inocula were kept on ice at all times. Replicators with 96 one-millimeter pins (Boekel Industries no. 140500) were sterilized in an autoclave before the start of the experiment and with an open flame between transfer steps. The use of multiple replicators ensured that the transfer pins had cooled down to ambient temperature before every transfer performed during the experiment. The 96-pin replicators were used to transfer 1-μL aliquots of bacterial suspension from the inocula-containing 96-well deep well plates to the agar surface of the assay plates containing test article or control antibiotic. The same method was used to inoculate compound-free agar plates for growth control. Plates were incubated for up to 48 h at 36 ± 1°C in 5% CO_2_. After the incubation period, assay and growth control plates were inspected visually, and the MIC was recorded as the lowest concentration of compound that completely inhibited growth.

**(ii) ESKAPE and bacterial select agents.** E. faecium and Staphylococcus epidermidis were grown in BBL Muller Hilton II cation-adjusted broth (Becton, Dickinson and Company no. 212322) with the addition of 1% IsoVitaleX (Becton, Dickinson and Company no. 211875). F. tularensis was grown in Cysteine Heart Broth [10 g BBL brain heart infusion (Becton, Dickinson and Company no. 211059), 10 g proteose peptone (Sigma F29185), 10 g dextrose (Sigma D9434), 5 g sodium chloride (Sigma S3014), and 1 g l-cysteine (Sigma C7352) in 1 L water. All of the other bacterial strains were grown in BBL Muller Hilton II broth cation-adjusted (MH). Antibacterial susceptibility testing for these strains was performed following CLSI documents M07-11 (Methods for Dilution Antimicrobial Susceptibility Tests for Bacteria That Grow Aerobically, 11th edition) and M11-09 (Methods for Antimicrobial Susceptibility Testing of Anaerobic Bacteria, 9th edition).

### Microsomal stability assay.

Liver microsome stability assays were performed by BioDuro Inc. Human and mouse (CD-1 male) liver microsomes were used. Briefly, 2.5 μL of control and test compounds (dissolved and diluted in DMSO to 100 μM concentration) were added to 197.5 μL of reaction buffer (0.05 M phosphate reaction buffer [pH = 7.4]) and vortexed. Next, 50 μL of reaction buffer was added to solution and mixed via pipetting up and down 6 times. At each time point of 0, 30, and 60 min, an aliquot of 20 μL was removed from each tube. Liver microsomes (LM) working solution was prepared in 0.05 M phosphate reaction buffer (pH = 7.4) to a final concentration of 1.27 mg/mL. NADPH solution was prepared in 0.05 M phosphate buffer to afford a 5 mM buffer solution. Then, 2.5 μL of positive control (5× mixed) and test compounds were added into 197.5 of LM working solution. After vortexing and incubating for 5 min (37°C), 50 μL of NADPH solution was added and mixed via pipetting. At each time point of 0, 5, 15, 30, and 60 min, an aliquot of 20 μL was removed from each tube. Next, 250 μL of quenching solution was aliquoted to quench the reaction, which was then vortexed for 1 min and placed on ice for 40 min. After centrifugation at 4,000 × *g* for 15 min, 100 μL of supernatant was transferred using a multichannel pipette to 0.65-mL tubes. Samples were diluted with MeOH: H_2_O (1:1) as necessary. Samples were analyzed by high-pressure liquid chromatography coupled to tandem mass spectrometry (LC/MS/MS) performed on a Sciex Applied Biosystems Qtrap 4000 triple-quadrupole mass spectrometer coupled to a Shimadzu HPLC system to quantify the remaining compound. Data were processed using Analyst software (version 1.4.2; Applied Biosystems Sciex). Calculations were performed according to the formula CL_int_ (μL/min/mg protein) = ln 2 × 1,000/t_1/2_/protein conc., where the units for t_1/2_ are min and those for protein conc. are mg/mL. Metabolite identification on the microsomal extracts was performed using a Q-Exactive HRMS coupled with an Ultimate 3000 HPLC system and a Kinetix C18 2.1 × 50-mm 2.6-μm HPLC column.

### Animal models.

**(i) N. gonorrhoeae vaginal infection models.** Four dosing regimens of JSF-2659 were tested as described in Supplemental Fig. S1. A total of 60 female 6- to 7-week old BALB/c mice (6 groups; *n* = 8 to 10 mice/group) were implanted with a 21-day slow-release, 5-mg 17β estradiol pellet (Innovative Research of America) under the skin (day −4) to induce susceptibility to N. gonorrhoeae. Antibiotics (streptomycin [STM], trimethoprim sulfate [TMP]) were administered to suppress the overgrowth of commensal flora that occurs under the influence of estradiol (18, 42). Mice were inoculated on day −2 (2 days after estradiol treatment was initiated) with a dose of the challenge strain that infects 80 to 100% of mice (ID_80–100_) (10^4^ CFU for strain H041 [STM^R^], a streptomycin-resistant derivative of the multidrug-resistant strain H041, referred to as H041 for brevity) (19). Vaginal mucus was quantitatively cultured for N. gonorrhoeae on the mornings of the next two consecutive days (days −1 and 0) to confirm infection. On the day of treatment (day 0), JSF-2659 was solubilized in 0.5% carboxymethyl cellulose sodium salt (CMC; MP Bio) and 0.5% Tween 80 (Thermo Fisher) in sterile endotoxin-free water; this vehicle solution was also used as the negative control. On the afternoon of day 0 (and following the morning culture), JSF-2659 was administered orally as either a single dose (QD) or three doses every 6 h (TID) over a 24-h period. The vehicle control was administered TID. The positive control established for H041 was 5 doses of 48 mg/kg gentamicin (GEN) administered once daily for 5 days IP (0.2 mL), with treatment beginning on day 0 (43). Experimental groups were designated as outlined in Supplemental Fig. S1. Vaginal mucus was quantitatively cultured for N. gonorrhoeae for 8 consecutive days after treatment (days +1 through +8). Vaginal material was collected by wetting a Puritan rayon swab in sterile, endotoxin-free phosphate-buffered saline (PBS), gently inserting the swab into the vagina, and suspending the swab in 1 mL GC Medium Base broth (GCB). Broth suspensions were diluted in GCB (1:100 for H041), and diluted and undiluted samples were cultured on GC-VCNTS (GC-vancomycin, colistin, nystatin, trimethoprin sulfate, streptomycin) agar using the Autoplater automated plating system (Spiral Biotech). GC-VCNTS agar contained vancomycin, colistin, nystatin, trimethoprim sulfate (VCNT supplement; Difco BD, Product number 202408) and 100 μg/mL streptomycin sulfate. A portion of the swab was also inoculated onto heart infusion agar to monitor the presence of facultative aerobic commensal flora. The number of viable N. gonorrhoeae bacteria recovered was determined using the Spiral Biotech Q-Counter Software at 48 h of incubation at 37°C. The percentage of mice in each test group that were culture-positive at each time point were plotted as Kaplan Meier colonization curves and compared to the positive control and vehicle control and to the other test groups. Differences were analyzed by the log-rank (Mantel-Cox) test. Colonization load, defined as the number of CFU per mL of vaginal swab suspension, were also compared among groups using a repeated-measures analysis of variance (ANOVA) with Bonferroni multiple comparisons. *P* values of <0.05 were considered significant. Mice must have had at least 3 consecutive days of negative culture to be considered cleared of infection. At the study endpoint, mice were euthanized using compressed CO_2_ gas in a CO_2_ gas chamber in the Laboratory Animal Medicine Facility. All animal experiments were conducted at the Uniformed Services University of the Health Sciences, a facility fully accredited by the Association for the Assessment and Accreditation of Laboratory Animal Care, under a protocol approved by the USUHS Institutional Animal Care and Use Committee, in accordance with all applicable federal regulations governing the protection of animals in research.

A secondary model (Pharmacology Discovery Services Taiwan, Ltd.) was performed according to the methods of Song et al. (44). A vaginal infection model using N. gonorrhoeae strain F1090 (ATCC 700825) was performed with groups of 5 ovariectomized BALB/c mice aged 5 to 6 weeks. Ovariectomy was performed at 4 weeks of age. The period of surgical recovery and acclimation was ~7 days. Animals were subcutaneously (SC) injected with 17β-estradiol solution solubilized in cotton seed oil at 0.23 mg/mouse 2 days before infection (day −2) and on the day of infection (day 0). To minimize the commensal vaginal bacteria, animals were treated twice daily with streptomycin (1.2 mg/mouse) and vancomycin (0.6 mg/mouse) by IP injection, along with trimethoprim sulfate at 0.4 mg/mL supplied in the drinking water. Antibacterial treatments were started 2 days prior to infection and continued daily until the end of study. On day 0, animals were inoculated intravaginally (IVG) with N. gonorrhoeae under anesthesia with IP injection of pentobarbital (80 mg/kg). The vagina was rinsed with 50 mM HEPES (30 μL [pH 7.4]) followed by inoculation with N. gonorrhoeae ATCC 700825 suspension at 0.02 mL/mouse. The target inoculation density was 2.0 × 10^6^ CFU/mouse and the actual count was 1.14 × 10^6^ CFU/mouse. A 0.05-mL aliquot was inoculated into a chocolate agar plate and incubated at 35 to 37°C with 5% CO_2_ overnight. The culture was resuspended in 1 mL PBS (>1.0 × 10^10^ CFU/mL, optical density at 620 nm [OD_620_]: 2.0 to 2.2) and diluted in PBS containing 0.5 mM CaCl_2_ and 1 mM MgCl_2_ to obtain the target inoculum of 1.0 × 10^8^ CFU/mL. Colony counts were determined by plating dilutions to chocolate agar plates followed by 20 to 24 h of incubation. The actual CFU count was 5.7 × 10^7^ CFU/mL. JSF-2659, at 75 and 250 mg/kg, was administered orally with two dosing schedules, including once (QD) at 2 h after infection and three times with a 6-h interval at 2, 8, and 14 h after infection. Test articles were also administered intramuscularly with JSF-2659 at 75 and 250 mg/kg once at 2 h after infection. Two reference compounds were applied in this study, ciprofloxacin and gentamicin. Ciprofloxacin was administered orally at 12.5 mg/kg once at 2 h after infection. Gentamicin was administered intraperitoneally at 48 mg/kg once at 2 h after infection for 1, 3, or 7 consecutive days depending on the time point of animal scarification, at 26, 74 or 170 h after infection. One infected but untreated group was euthanized at 2 h after infection for the initial bacterial counts. Each animal was weighed prior to each dose, and the dose volumes were 10 mL/kg for all dosing groups. Test animals were euthanized by CO_2_ asphyxiation and euthanized at 26, 74, or 170 h after infection. Vaginal lavage was performed twice with 200 μL GC broth containing 0.05% saponin to recover vaginal bacteria, and the lavage fluids were pooled in a total volume of 500 μL. The bacterial counts (CFU/mL) in lavage fluid were calculated and the percentage decrease relative to the vehicle control was calculated by the following formula: decrease (%) = [(CFU/mL of vehicle – CFU/mL of treatment)/(CFU/mL of vehicle)] × 100%. Statistical significance was assessed with a one-way ANOVA followed by Dunnett’s method using Prism GraphPad software version 5.0. A decrease in the bacterial counts of the treated animals compared to the vehicle control group where *P < *0.05 was considered significant. The animal study was performed in an AAALAC-accredited ABSL2 laboratory under the supervision of veterinarians, and the animal care and use protocol was approved by the IACUC at Pharmacology Discovery Services Taiwan, Ltd.

**(ii) Staphylococcus soft tissue infection model.** A deep tissue thigh model of S. aureus was contracted to Pharmacology Discovery Services Taiwan, Ltd. All studies were performed with S. aureus strain VRS-2: a VRSA strain Hershey, Van-A producing SCC Mec II, st5 strain that was isolated from the foot ulcer of a 70-year-old patient. It is methicillin-resistant with resistance to carbapenems, cephalosporins, and penicillins. VRS-2 is resistant to vancomycin (MIC > 64 μg/mL), quinolones (LVX-R, CIP-R), macrolides (ERY-R, CLI-R), and trimethoprim-sulfamethoxazole. Groups of 5 male or female specific-pathogen-free ICR mice weighing 22 ± 2 g were used. Animals were immunosuppressed by two intraperitoneal injections of cyclophosphamide: the first at 150 mg/kg 4 days before infection (day −4) and the second at 100 mg/kg 1 day before infection (day −1). On day 0, animals were inoculated intramuscularly (0.1 mL/thigh) with vancomycin resistant (VRS-2) S. aureus into the right thigh. Vehicle and/or test substances, including linezolid at 50 mg/kg BID, were then administered (SC or PO) 2 and 14 h later. A single vehicle dosing group was used as a comparator for this study. Prior studies have shown no changes in thigh burdens from different doses or administration routes of the vehicle and meet the requirement of reduction of animals per IACUC policy. At 24 h after treatment, animals were humanely euthanized with CO_2_ asphyxiation and then the muscle of the right thigh was harvested from each test animal. The removed muscle tissues were homogenized in 3 mL of PBS [pH 7.4] with a polytron homogenizer. Homogenates, 0.1 mL, were used for serial 10-fold dilutions and plated on nutrient agar plates for colony counts. The animal study was performed in an AAALAC-accredited ABSL2 laboratory under the supervision of veterinarians, and the animal care and use protocol was approved by the IACUC at Pharmacology Discovery Services Taiwan, Ltd.

**(iii) Hamster colitis model.** This model contracted to Pharmacology Discovery Services Taiwan, Ltd. assessed test articles for protection against a lethal C. difficile colitis infection (45). Groups of 10 male or female Golden Syrian hamsters weighing 90 ± 10 g, provided by the National Laboratory Animal Center (Taiwan), were used. Each animal was pretreated with a single subcutaneous dose of clindamycin at 50 mg/kg (day −1) to induce susceptibility to C. difficile. Twenty-four hours after the clindamycin treatment (day 0), the animals were infected with C. difficile BAA-1805, a ribotype 027 strain. Spores were administered in a single oral lethal (LD_90–100_) dose of 1 × 10^5^ spores per animal. Test substance and vehicle were administered (PO, IP, IV, or SC) 16 h after inoculation, then twice daily for a total of 5 consecutive days. Mortality was recorded daily for 14 days following infection. Prevention of mortality in 50% or more of the animals indicated significant activity. The C. difficile strain BAA-1805, a toxigenic, NAP1, Ribotype 027 strain, was obtained from the American Type Culture Collection (Rockville, MD) and cryopreserved as single-use frozen working stock cultures stored at −80°C. A 0.1-mL aliquot was transferred to anaerobic blood agar plates and incubated in an anaerobic workstation (Don Whitley A35) at 35°C to 37°C anaerobic condition (80% N_2_, 10% CO_2_, 10% H_2_) for 5 days. Growth on plates was transferred to phosphate-buffered saline and heated at 70°C for 30 min to inactivate the spores. The heated culture was pelleted by centrifugation at 3,500 × *g* for 15 min, and then resuspended in cold PBS (>5 × 10^7^ spores/mL in original). The culture was diluted in PBS to an estimated concentration of 0.6 to 1.0 × 10^6^ spores/mL. The actual colony counts were determined by plating dilutions onto CCFA-HT (cycloserine cefoxitin fructose agar with horse blood and taurocholate) plates, followed by 2 days incubation and colony counting. The actual inoculum was 1.16 × 10^6^ spores/mL. Animals were individually housed in animal cages. All animals were maintained in a well-controlled temperature (20°C to 24°C) and humidity (30% to 70%) environment with 12-h light/dark cycles. Free access to standard lab diet (MFG [Oriental Yeast Co., Ltd., Japan]) and autoclaved tap water were granted for the study period. All aspects of this work, including housing, experimentation, and animal disposal, were performed in general accordance with the Guide for the Care and Use of Laboratory Animals (National Academies Press, Washington, DC, 2011). The study was performed in our AAALAC-accredited ABSL2 laboratory under the supervision of staff veterinarians. The animal care and use protocol was approved by the IACUC of Pharmacology Discovery Services Taiwan, Ltd.

### Quantitative methods.

Levels of JSF-2414 in plasma and tissues were measured by LC-MS/MS in electrospray positive-ionization mode (ESI+) on a Sciex Qtrap 4000 triple-quadrupole mass combined with an Agilent 1260 HPLC using Analyst software and multiple-reaction monitoring (MRM) of precursor/product transitions. The following MRM transitions were used for JSF-2414 (494.28/324.80) and the internal standard Verapamil (455.4/165.2). Chromatography was performed with an Agilent Zorbax SB-C8 column (2.1 × 30 mm; particle size: 3.5 μm) using a reverse phase gradient elution. Here, 0.1% formic acid in Milli-Q deionized water was used for the aqueous mobile phase and 0.1% formic acid in acetonitrile (ACN) was used for the organic mobile phase. Tissues were homogenized prior to extraction by combining 4 parts PBS buffer with 1 part tissue. The samples were homogenized using a SPEX Sample Prep Geno/Grinder 2010 for 5 min at 1,500 × *g*. 1 mg/mL DMSO stock was serially diluted in 50/50 ACN/water to create standard curves and quality control spiking solutions. Twenty μL of neat spiking solutions was added to 20 μL of drug-free mouse K2EDTA plasma (Bioreclamation) or drug-free tissue homogenate, and extraction was performed by adding 200 μL of acetonitrile/methanol 50/50 protein precipitation solvent containing 10 ng/mL verapamil (Sigma-Aldrich). Extracts were vortexed for 5 min and centrifuged at 4,000 × *g* for 5 min. The supernatants were analyzed by LC-MS. Sample analysis was accepted if the concentrations of the quality control samples were within 20% of the nominal concentration.
