# Supplementary material for: A Novel Oral GyrB/ParE Dual Binding Inhibitor Effective against Multidrug-Resistant Neisseria gonorrhoeae and Other High-Threat Pathogens
Source: Antimicrob Agents Chemother. 2022 Aug 16;66(9):e00414-22. doi: 10.1128/aac.00414-22 (PMC9487510; doi:10.1128/aac.00414-22)
Supplement: Supplemental file 1 — Supplemental methods, Fig. S1 and S2, and Tables S1 and S2. Download aac.00414-22-s0001.pdf, PDF file, 0.8 MB [file aac.00414-22-s0001.pdf]

SUPPLEMENT

Figure Legends

Figure S1. Experimental design schematic of the *Neisseria gonorrhoeae* infection model with strain H041. The following test regimens were performed: JSF-2659; Group 1: 75 mg/kg, PO, 1 dose (QD), 10 mL/kg; Group 2: 75 mg/kg, PO, 3 doses every 6 h (TID), 10 mL/kg; Group 3: 250 mg/kg, PO, 1 dose (QD), 10 mL/kg; Group 4: 250 mg/kg, PO, 3 doses every 6 h (TID),10 mL/kg; Positive control group Group 5: GEN, 48 mg/kg, 5 doses once daily, IP, 0.2 mL and Vehicle control group Group 6: 0.5% CMC/0.5% Tween 80, 3 doses (TID), 10 mL/kg

Figure S2. Individual mouse vaginal swab data points from the JSF-2659 efficacy study against the multi-drug resistant *Neisseria gonorrhoeae* strain H041 in the murine vaginal infection model. Ten mice per group were used and each mouse is represented by the shape and color listed: black circle, green triangle, red triangle, blue diamond, orange circle, purple triangle black diamond, yellow circle, gray square and red diamond. The treatment groups included: Vehicle control group: 0.5% CMC/0.5% Tween 80, 3 doses (TID), 10 mL/kg; Positive control group: GEN, 48 mg/kg, 5 doses once daily, IP, 0.2 mL; 75 mg/kg JSF-2659 group: PO, 3 doses every 6 h (TID), 10 mL/kg; 250 mg/kg JSF-2659 group: PO, 3 doses every 6 h (TID),10 mL/kg.

Supplement Methods

Preparation of JSF-2414 and JSF-2659. It should be noted that JSF-2414 and JSF-2659 were prepared as single enantiomers utilizing chiral (-)-(2-oxa-8-azaspiro[4.5]decan-3-yl)methanol. While the absolute stereochemistry of this building block was not established, its optical rotation was measured as $[\alpha]_D^{22}$ –3.14 (*c* 0.1, MeOH). The molecules are shown with a defined stereochemistry (arbitrarily as *R*).

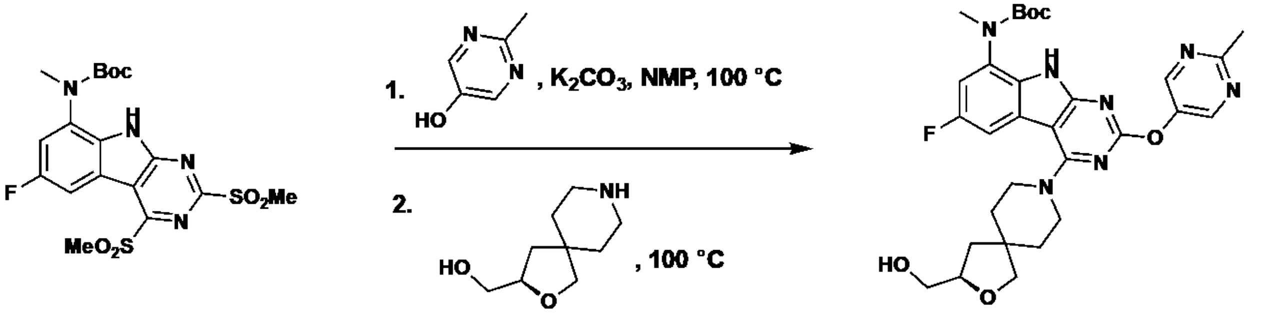

A1 A2

To *tert*-butyl (6-fluoro-2,4-bis(methylsulfonyl)-9H-pyrimido[4,5-b]indol-8-yl)(methyl)carbamate (9.45 g, 20.0 mmol; Source: WuXiAppTec) in NMP (50 mL) was added 2-methylpyrimidine-5-ol (8.80 g, 80.0 mmol) and potassium carbonate (11.00 g, 79.7 mmol). The mixture was heated at 100 °C for 70 min, then (-)-(2-oxa-8-azaspiro[4.5]decan-3-yl)methanol hydrogen chloride salt(4.15 g, 20.0 mmol; Source: WuXiAppTec; $[\alpha]_{D}22$ = -3.14 (c 0.1, MeOH)) was added. The mixture was heated at 100 °C for 2.5 h. 400 mL water was added with stirring, after which the precipitate was filtered and washed with water to give *tert*-butyl (6-fluoro-4-(3-(hydroxymethyl)-2-oxa-8-azaspiro[4.5]decan-8-yl)-2-((2-methylpyrimidin-5-yl)oxy)-9H-pyrimido[4,5-b]indol-8-yl)(methyl)carbamate as a yellow solid in 71.5% yield (8.50 g, 14.3 mol). The product was used in the next step without further purification.

Scheme 2

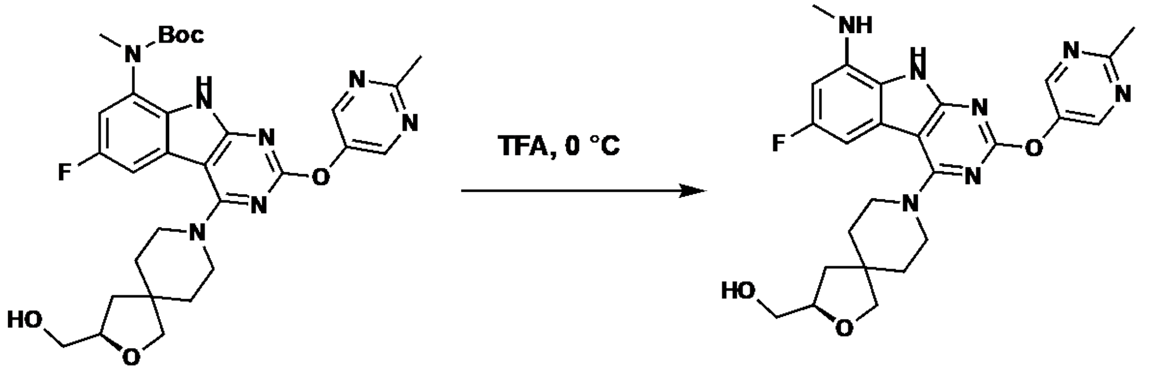

A2 JSF-2414

A mixture of A2 = *tert*-butyl (6-fluoro-4-(3-(hydroxymethyl)-2-oxa-8-azaspiro[4.5]decan-8-yl)-2-((2-methylpyrimidin-5-yl)oxy)-9H-pyrimido[4,5-b]indol-8-yl)(methyl)carbamate (3.00 g, 5.05 mmol) in 12 mL TFA was stirred at 0 °C for 1 min. The

mixture was concentrated *in vacuo*, after which it was diluted with 10 mL ethanol and 20 mL water. The pH of the mixture was adjusted to 10 by adding 6 N NaOH(aq).The resulting mixture was stirred for 8 h and was then poured into 50 mL saturated NH4Cl(aq). The mixture was extracted with ethyl acetate (4x50 mL). The combined extracts were diluted to 100 mL and washed with saturated aqueous brine solution. The organic layer was dried and concentrated under reduced pressure. Purification by flash column chromatography on silica gel, eluting with 0-5% methanol/dichloromethane provided JSF-2414 as a yellow solid in 73.9% yield (1.84 g, 3.73 mol). ^{1}H NMR (500 MHz, d_6-DMSO) δ 11.7 (s, 1), 8.73 (s, 2), 6.63 (dd, J = 10.0, 1.3 Hz, 1), 6.35 (dd, J = 12.1, 1.4 Hz, 1), 5.58 (d, J = 2.9 Hz, 1), 4.67 (s, 1), 4.04 – 3.90 (m, 1), 3.68 (ddd, J = 18.1, 13.0, 5.1 Hz, 2), 3.63 – 3.47 (m, 4), 3.41 (d, J = 4.7 Hz, 2), 2.85 (d, J = 3.8 Hz, 3), 2.66 (s, 3), 1.93 (dd, J = 12.3, 7.2 Hz, 1). $[\alpha]_D22$ = -3.49 (c 0.5, MeOH). LRMS m/z: $[M+H]^+$Calcd for $C_{25}H_{29}FN_7O_3$ 494.2; found 494.2.

Scheme 3

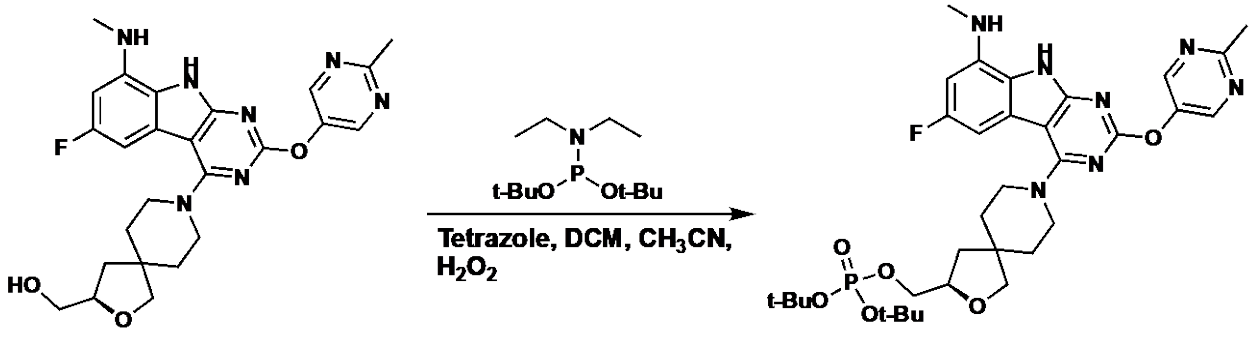

JSF-2414 **A3**

To JSF-2414 (1.45 g, 2.94 mmol) in dichloromethane (20 mL) and acetonitrile (2 mL) was added a tetrazole solution (18.0 mL, 8.10 mmol, 0.45 M in acetonitrile) and di-*tert*-butyl N,N-diethylphosphoramidite (2.20 mL, 7.91 mmol). The mixture was stirred at rt for 2 h. Then a 20% aqueous solution of H_2O_2 (3.0 mL) was added. After being stirred at rt for 10 min, the mixture was extracted with ethyl acetate. The organic phase was washed with saturated aqueous brine solution and dried over anhydrous sodium sulfate. The

solution was filtered, concentrated and purified by flash column chromatography on silica gel, eluting with 0 – 4% methanol/dichloromethane to give di-*tert*-butyl ((8-(6-fluoro-8-(methylamino)-2-((2-methylpyrimidin-5-yl)oxy)-9H-pyrimido[4,5-b]indol-4-yl)-2-oxa-8-azaspiro[4.5]decan-3-yl)methyl) phosphate as a brown oil in 64.6% yield (1.30 g, 1.90 mmol). LRMS m/z: $[M+H]^+$Calcd for $C_{33}H_6FN_7O_3P$ 686.3; found 686.2.

Scheme 4

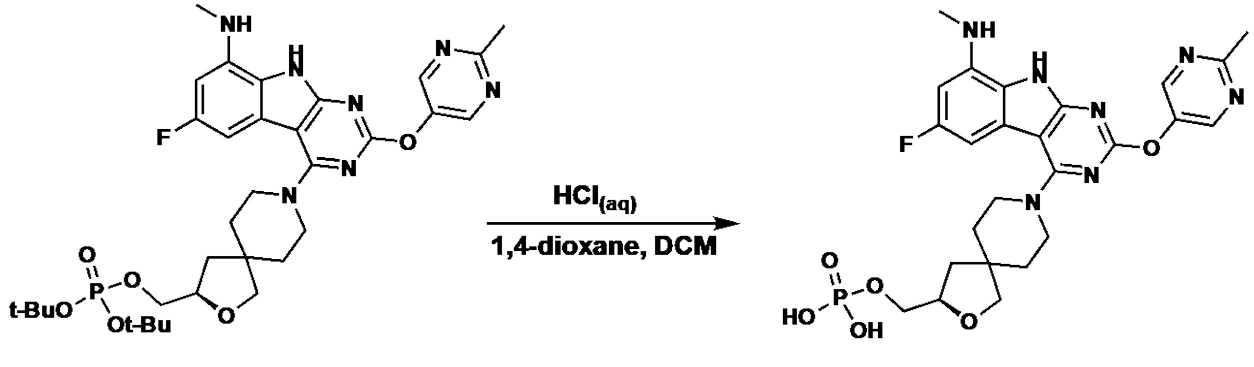

A3 **JSF-2659**

To A3 = di-*tert*-butyl ((8-(6-fluoro-8-(methylamino)-2-((2-methylpyrimidin-5-yl)oxy)-9H-pyrimido[4,5-b]indol-4-yl)-2-oxa-8-azaspiro[4.5]decan-3-yl)methyl) phosphate (260 mg, 0.379 mmol) in dichloromethane (10 mL) was added 0.7 mL 4 N $HCl_{(aq)}$ in 1,4-dioxane (2.8 mmol) dropwise. The mixture was stirred at rt for 10 min. Then dichloromethane was removed by pipette. The solid was washed with ethyl acetate (2 x 10 mL) and dried *in vacuo* to give JSF-2659 as a yellow powder in 99% yield (250 mg, 0.377 mmol). Elemental analysis was consistent with two HCl and one H_2O molecules per molecule of targeted product. ^{1}H NMR (500 MHz, D_2O) δ 8.53 (s, 2), 6.27 (d, J = 11.8 Hz, 1), 6.22 (d, J = 10.2 Hz, 1), 4.26 – 4.14 (m, 1), 3.82 – 3.72 (m, 1), 3.70 – 3.61 (m, 1), 3.58 (s, 2), 3.29 – 3.05 (m, 4), 2.75 (s, 3), 2.62 (s, 3), 1.97 – 1.85 (m, 1), 1.46 (s, 5). Two Hs were unaccounted for and presumably were the two N-Hs which were exchanging with D2O. LRMS m/z: $[M+H]^+$Calcd for $C_{25}H_{29}FN_7O_6P$ 573.2; found 574.2. The bis-sodium salt JSF-2659-B was prepared by adding saturated aqueous NaHCO3 to the HCl salt in water. Purification by HPLC, eluting with water/acetonitrile gave a white solidJSF-2659-B. 1H NMR (500 MHz, d6-DMSO) δ 11.9 (s, 1), 8.76 (s, 2), 6.67 (d, J = 9.8 Hz, 1), 6.41 (d, J = 11.8, 1), 4.10 – 4.10 (m, 1), 3.87 – 3.77 (m, 2), 3.75 – 3.62 (m, 2), 3.59 (s, 3), 3.57 – 3.49 (m, 2), 2.85 (s,

3), 2.67 (s, 3), 1.73 – 1.61 (m, 4), 1.59(s, 1), 1.53 (dd, J = 12.2, 8.6 Hz, 1). Also noted 10.3– 9.0 (brs, solvent H2O bound to OH of acid). LRMS m/z: $[M+H]^+$Calcd for $C_{25}H_{30}FN_7O_6P$ 574.2; found 574.2.

hERG inhibition assay. These assays were conducted by BioDuro, Incorporated. HEK-293 cells stably expressing the hERG K^+ channel were sourced from the Institute of SARL (CreaCell). Cells were grown in DMEM supplemented with 10 % FBS and 0.8 mg/mL G418. For electrophysiology assays, the cells were continuously superfused by extracellular saline containing 140 mM NaCl, 3.5 mM KCl, 1 mM $MgCl_2$, 2 mM $CaCl_2$, 10 mM dextrose, 10 mM HEPES, and 1.25 mM NaH_2PO_4, pH 7.4. Test compounds were dissolved in DMSO at a concentration of 10 mM for cisapride, a positive control (IC_{50} = 18.0 ± 0.92 nM), and 30 mM for JSF-2414 and JSF-2659 and diluted in extracellular saline to the appropriate concentration (1 nM – 1 μM for cisapride and 0.3 – 30 μM for the test articles). The glass micropipettes for whole-cell patch-clamp recording were filled with intracellular saline with 20 mM KCl, 115 mM potassium L-aspartate, 1 mM $MgCl_2$, 5 mM EGTA, 10 mM HEPES, 2 mM Na_2-ATP, pH 7.2.

The hERG current was quantified at a holding potential of -80 mV and then depolarized to -50 mV for 0.5 s to assess the leak current. The voltage was then depolarized to 30 mV for 2.5 s. The peak tail current was induced by a repolarizing pulse to -50 mV for 4 s. An inter-pulse interval of 10 s enabled recovery from inactivation to measure the effect of the test compound on the hERG tail current. The test article was then supplied to the investigated cell from a nearby capillary, usually for another three min per concentration. The data were collected by EPC-10 amplifier and stored in PatchMaster (HEKA) software. Current amplitude values were graphed versus time to depict the effect of the compound and quantify the percentage of channel inhibition. The results from at least three cells were pooled and fitted with a non-linear regression to estimate the mean IC_{50} value.

Mouse Pharmacokinetics studies. All animal studies were ethically reviewed and carried out in accordance with the Hackensack Meridian Health Institutional Animal Care and Use Committee. Six-week-old CD-1 female mice (20–25 g) were used in pharmacokinetic studies. Groups of 3 mice received a single oral or IM dose of JSF 2659

formulated in 0.4% methylcellulose, at either 5, or 25 mg/kg. Aliquots of 20 µl of blood were collected from the lateral tail vein by serial puncture from each mouse at 0.5, 1, 3, 5, 7 and 24h post dose in K2EDTA tubes. Plasma was recovered after centrifugation and stored at −80°C until analyzed by high pressure liquid chromatography coupled to tandem mass spectrometry.

Supplemental Table 1. Individual MIC values for clinical isolates of *Neisseria gonorrhoeae*.

Strain ID		Test and Control Articles MIC (µg•ml^{-1})						
Southern Research	CDC	JSF-2414	Azi	Cfx	Cro	Cip	Pen	Tet
		MIC Range	MIC Range	MIC Range	MIC Range	MIC Range	MIC Range	MIC Range
		**0.0005-0.03**	**0.016->16.0**	**0.016 – 4**	**<0.002-0.125**	**<0.016 – 16**	**<0.008 – 4**	**0.063 – 16**
**CDC1**	**GCREF2012016**	0.0039	0.25	0.125	0.031	8	1	2
**CDC2**	**GCREF2012045**	0.0039	0.5	0.125	0.031	16	1	1
**CDC3**	**GCREF2013013**	0.0039	0.125	0.125	0.031	8	1	1
**CDC4**	**GCREF2013030**	0.0039	0.125	0.125	0.031	8	1	0.5
**CDC5**	**GCREF2012001**	0.0039	0.5	0.125	0.063	8	1	1
**CDC6**	**GCREF2012002**	0.0078	0.5	0.25	0.063	8	1	1
**CDC8**	**GCREF2012004**	0.002	0.125	0.125	0.016	8	0.25	1
**CDC9**	**GCREF2012005**	0.0039	0.125	0.125	0.031	8	1	1
**CDC10**	**GCREF2012006**	0.0039	0.5	0.125	0.031	16	1	1
**CDC11**	**GCREF2012007**	0.0039	0.25	0.125	0.031	8	1	1
**CDC12**	**GCREF2012008**	0.0039	0.125	0.25	0.031	8	1	1
**CDC13**	**GCREF2012009**	0.0039	0.125	0.063	0.031	8	1	1
**CDC14**	**GCREF2012010**	0.0039	0.125	0.25	0.031	8	1	1
**CDC15**	**GCREF2012011**	0.0078	4	0.031	0.008	2	0.125	0.5
**CDC16**	**GCREF2012012**	0.0078	0.25	4	0.063	8	2	1

**CDC18**	**GCREF2012 014**	0.0078	0.5	0.063	0.063	16	1	1
**CDC19**	**GCREF2012 015**	0.0039	8	0.125	0.008	<0.016	0.25	0.5
**CDC20**	**GCREF2012 017**	0.016	>16.0	0.125	0.063	2	0.125	1
**CDC21**	**GCREF2012 018**	0.0039	0.125	0.25	0.031	16	1	1
**CDC22**	**GCREF2012 019**	0.03	0.5	0.031	0.008	4	0.25	1
**CDC23**	**GCREF2012 020**	0.0078	0.125	0.125	0.031	8	1	1
**CDC24**	**GCREF2012 021**	0.0039	0.25	0.063	0.063	8	1	1
**CDC25**	**GCREF2012 022**	0.0039	0.125	0.25	0.031	8	2	1
**CDC26**	**GCREF2012 023**	0.0078	0.5	0.063	0.031	4	0.25	0.5
**CDC27**	**GCREF2012 024**	0.0039	0.25	0.063	0.031	8	1	1
**CDC28**	**GCREF2012 025**	0.0039	0.25	0.25	0.031	16	1	1
**CDC29**	**GCREF2012 026**	0.0078	0.5	0.125	0.016	<0.016	1	1
**CDC30**	**GCREF2012 027**	0.0078	0.25	0.125	0.031	8	1	1
**CDC31**	**GCREF2012 028**	0.0078	0.5	0.25	0.031	8	2	2
**CDC32**	**GCREF2012 029**	0.0078	0.125	0.125	0.063	8	1	0.5
**CDC33**	**GCREF2012 030**	0.0078	0.125	0.5	0.125	2	1	0.5
**CDC34**	**GCREF2012 031**	0.0039	0.125	0.063	0.125	16	1	1
**CDC35**	**GCREF2012 032**	0.0039	0.25	0.25	0.125	16	2	2
**CDC36**	**GCREF2012 033**	0.002	4	0.063	0.031	8	0.25	1
**CDC37**	**GCREF2012 034**	0.0039	0.25	0.125	0.031	16	1	1
**CDC38**	**GCREF2012 035**	0.0039	0.25	0.063	0.031	16	1	1
**CDC39**	**GCREF2012 036**	0.0039	0.25	0.25	0.031	8	2	1
**CDC40**	**GCREF2012 037**	0.0039	0.25	0.125	0.031	8	1	1
**CDC41**	**GCREF2012 038**	0.0039	0.25	0.25	0.031	8	2	1

**CDC42**	**GCREF2012 039**	0.0039	0.25	0.25	0.031	16	2	2
**CDC43**	**GCREF2012 040**	0.0039	0.25	0.25	0.031	16	2	2
**CDC45**	**GCREF2012 042**	0.0078	0.5	0.25	0.125	16	2	2
**CDC46**	**GCREF2012 043**	0.0039	0.125	0.125	0.031	16	1	1
**CDC47**	**GCREF2012 044**	0.0039	0.5	0.125	0.031	16	1	1
**CDC48**	**GCREF2012 046**	0.0039	0.25	0.125	0.031	8	1	1
**CDC49**	**GCREF2012 047**	0.0039	0.5	0.25	0.031	16	2	1
**CDC50**	**GCREF2012 048**	0.0078	0.25	0.25	0.063	16	2	1
**CDC51**	**GCREF2012 049**	0.0039	0.125	0.125	0.008	16	2	1
**CDC52**	**GCREF2012 050**	0.0039	0.125	0.25	0.008	16	2	1
**CDC53**	**GCREF2012 051**	0.0078	0.125	0.25	0.004	16	2	1
**CDC54**	**GCREF2012 052**	0.0039	0.016	0.25	0.004	16	2	1
**CDC55**	**GCREF2013 001**	0.0078	0.125	0.125	0.031	16	1	1
**CDC56**	**GCREF2013 002**	0.0039	0.125	0.25	0.016	16	1	1
**CDC57**	**GCREF2013 003**	0.0039	0.125	0.125	0.004	<0.016	1	0.5
**CDC58**	**GCREF2013 004**	0.002	0.125	0.25	0.004	16	2	1
**CDC59**	**GCREF2013 044**	0.002	0.125	0.25	0.004	4	2	16
**CDC60**	**GCREF2013 005**	0.0078	0.125	0.125	0.031	16	4	1
**CDC61**	**GCREF2013 006**	0.0078	0.25	0.125	0.031	32	4	1
**CDC62**	**GCREF2013 007**	0.016	0.125	0.25	0.008	0.125	0.25	0.5
**CDC63**	**GCREF2013 008**	0.0078	0.016	0.125	<0.002	<0.016	<0.008	0.063
**CDC64**	**GCREF2013 009**	0.016	0.125	0.125	0.008	<0.016	0.5	1
**CDC65**	**GCREF2013 010**	0.0078	0.125	0.125	0.016	<0.016	0.5	0.5
**CDC66**	**GCREF2013 011**	0.016	4	0.125	0.008	<0.016	0.25	0.5
**CDC67**	**GCREF2013 014**	0.0078	0.125	0.125	0.008	0.25	0.5	1

**CDC68**	**GCREF2013 016**	0.002	0.125	0.063	0.016	16	1	1
**CDC69**	**GCREF2013 017**	0.002	0.125	0.063	0.031	16	1	1
**CDC70**	**GCREF2013 018**	0.0039	4	0.125	0.004	16	2	1
**CDC71**	**GCREF2013 019**	0.016	0.125	0.016	0.008	0.25	0.25	1
**CDC72**	**GCREF2013 020**	0.0078	0.125	0.25	0.031	16	2	1
**CDC73**	**GCREF2013 021**	0.016	0.125	0.125	0.008	0.125	0.5	1
**CDC74**	**GCREF2013 022**	0.016	0.125	0.125	0.008	<0.016	0.25	1
**CDC75**	**GCREF2013 023**	0.0078	0.5	0.125	0.016	<0.016	0.5	1
**CDC76**	**GCREF2013 024**	0.0078	0.125	0.125	0.016	<0.016	0.5	1
**CDC77**	**GCREF2013 025**	0.0078	4	0.125	0.008	0.25	0.125	1
**CDC78**	**GCREF2013 026**	0.0005	0.031	0.063	0.016	2	0.5	1
**CDC79**	**GCREF2013 027**	0.0039	0.125	0.125	0.016	16	1	1
**CDC80**	**GCREF2013 028**	0.0039	8	0.125	0.008	0.25	2	1
**CDC81**	**GCREF2013 029**	0.0039	0.125	0.25	0.031	16	2	1
**CDC82**	**GCREF2013 031**	0.0078	2	0.125	0.031	32	1	0.5
**CDC83**	**GCREF2013 032**	0.016	0.125	0.125	0.008	0.125	0.25	0.5
**CDC84**	**GCREF2013 033**	0.0078	0.125	0.125	0.008	<0.016	0.25	0.5
**CDC86**	**GCREF2013 035**	0.0078	0.125	0.125	0.008	0.25	<0.008	0.25
**CDC87**	**GCREF2013 036**	0.0039	0.125	0.25	0.063	16	2	1
**CDC88**	**GCREF2013 037**	0.0078	2	0.125	0.031	16	2	0.5
**CDC89**	**GCREF2013 038**	0.0039	2	0.125	0.008	16	1	1
**CDC90**	**GCREF2013 039**	0.0078	0.125	0.125	0.031	16	2	1
**CDC91**	**GCREF2013 040**	0.0078	0.125	4	0.125	16	2	1
**CDC92**	**GCREF2013 041**	0.0039	0.125	0.125	0.031	16	2	1
**CDC93**	**GCREF2013 042**	0.0039	0.125	0.125	0.016	16	1	1

**CDC94**	**GCREF2013 043**	0.0078	2	0.125	0.008	<0.016	0.25	0.5
**CDC95**	**GCREF2013 045**	0.0078	0.125	0.25	0.031	16	2	1
**CDC96**	**GCREF2013 046**	0.002	0.125	0.125	0.016	16	1	0.5
**CDC97**	**GCREF2013 047**	0.0039	0.125	0.125	0.031	16	1	1
**CDC98**	**GCREF2013 048**	0.0078	2	0.125	0.008	<0.016	0.25	0.5
**CDC99**	**GCREF2013 012**	0.0078	4	0.125	0.008	<0.016	0.25	0.5
**CDC10 0**	**GCREF2013 015**	0.0039	0.125	0.25	0.031	16	1	1

Supplemental Table 2. Individual MIC values for clinically important Gram positive bacteria, .

		Test and Control Articles MIC (µg•ml-1)			
**Species**	**Strain#**	**CIP**	**RIF**	**VAN**	**JSF-2414**
MRSA	23617	0.125	ND	0.5	0.031
MRSA	23604	0.25	ND	0.5	0.031
MRSA	23590	0.062	ND	1.0	0.016
MRSA	23631	0.062	ND	1.0	0.031
MRSA	21357	0.25	ND	0.5	0.016
MRSA	21333	0.25	ND	0.5	0.016
MRSA	21184	>4	ND	0.5	0.031
MRSA	21319	0.5	ND	0.5	0.016
MRSA	21341	0.25	ND	ND	0.125
MRSA	21343	0.25	ND	ND	0.062
MRSA	21347	>4	ND	ND	0.125
MRSA	21353	>4	ND	ND	0.062
MRSA	21354	>4	ND	ND	0.062
MRSA	21355	1.0	ND	ND	0.062
MRSA	21356	>4	ND	ND	0.062
MRSA	21358	0.25	ND	ND	0.031
MRSA	21141	>4	ND	ND	0.062
MRSA	21143	>4	ND	ND	0.062
MRSA	21157	>4	ND	ND	0.125
MRSA	21165	>4	ND	ND	0.062
MRSA	21166	>4	ND	ND	0.062

MRSA	21171	>4	ND	ND	0.062
MRSA	21174	>4	ND	ND	0.062
MRSA	21179	>4	ND	ND	0.062
MRSA	21183	0.25	ND	ND	0.062
MRSA	21187	>4	ND	ND	0.062
MRSA	21190	>4	ND	ND	0.125
MRSA	21191	0.25	ND	ND	0.016
MRSA	21213	0.062	ND	ND	0.125
MRSA	21276	0.25	ND	ND	0.031
MRSA	21323	>4	ND	ND	0.125
MRSA	USA400Padm	>4	ND	ND	0.062
MRSA	21142	>4	ND	ND	0.016
MRSA	21156	>4	ND	ND	0.004
MRSA	21158	>4	ND	ND	0.002
MRSA	21160	>4	ND	ND	0.004
MRSA	21161	>4	ND	ND	0.004
MRSA	21167	>4	ND	ND	0.004
MRSA	21168	>4	ND	ND	0.004
MRSA	21175	>4	ND	ND	0.008
MRSA	21180	>4	ND	ND	0.002
MRSA	21186	>4	ND	ND	0.002
MRSA	21199	>4	ND	ND	0.008
MRSA	21278	0.25	ND	ND	0.016
MRSA	21288	0.25	ND	ND	0.004
MRSA	21326	0.25	ND	ND	0.004
MRSA	21327	>4	ND	ND	0.016
MRSA	21329	>4	ND	ND	0.002
MRSA	21332	>4	ND	ND	0.004
MRSA	21348	1.0	ND	ND	0.002
MRSA	21352	>4	ND	ND	0.002
MRSA	27862	>4	ND	ND	0.031
MRSA	27866	>4	ND	ND	0.031
MRSA	27917	>4	ND	ND	0.062
MRSA	27925	>4	ND	ND	0.031
MRSA	27933	0.25	ND	ND	0.031
MRSA	27965	>4	ND	ND	0.062
MRSA	27967	>4	ND	ND	0.031
MRSA	28036	0.5	ND	ND	0.062
MRSA	28046	>4	ND	ND	0.031
MRSA	28048	>4	ND	ND	0.031
MRSA	28062	0.5	ND	ND	0.031
MRSA	28154	0.125	ND	ND	0.031
MRSA	28513	>4	ND	ND	0.008

MRSA	28514	>4	ND	ND	0.031
MRSA	28754	0.25	ND	ND	0.031
MRSA	28755	0.25	ND	ND	0.031
MRSA	28777	0.5	ND	ND	0.016
MRSA	28847	0.5	ND	ND	0.016
MRSA	28871	0.5	ND	ND	0.016
MRSA	28883	>4	ND	ND	0.008
MRSA	28884	>4	ND	ND	0.004
MRSA	28913	>4	ND	ND	0.016
MRSA	28917	>4	ND	ND	0.008
MRSA	29062	>4	ND	ND	0.016
MRSA	27841	>4	ND	ND	0.062
MRSA	27896	>4	ND	ND	0.031
MRSA	27922	>4	ND	ND	0.031
MRSA	28067	2.0	ND	ND	0.062
MRSA	28512	>4	ND	ND	0.016
MRSA	28532	>4	ND	ND	0.062
MRSA	29155	>4	ND	ND	0.031
MRSA	29179	>4	ND	ND	0.016
MRSA	29306	>4	ND	ND	0.062
MRSA	29324	>4	ND	ND	0.031
MRSA	29086	0.25	ND	ND	0.016
MRSA	29098	0.5	ND	ND	0.031
MRSA	29184	0.5	ND	ND	0.031
MRSA	29330	1.0	ND	ND	0.031
MRSA	29695	1.0	ND	ND	0.031
MRSA	29709	>4	ND	ND	0.016
MRSA	26636	0.125	ND	ND	0.016
MRSA	26641	0.25	ND	ND	0.016
MRSA	27929	0.5	ND	ND	0.016
MRSA	29578	1.0	ND	ND	0.031
MRSA	30339	2.0	ND	ND	0.016
MRSA	36531	0.5	ND	ND	0.016
MRSA	36539	>4	ND	ND	0.016
MRSA	38043	0.5	ND	ND	0.002
MRSA	38068	1.0	ND	ND	0.008
MRSA	27967	>4	ND	ND	0.031
VRSA	VRS2	ND	>10	>200	0.031
VRSA	22523	ND	<0.078	100 - 200	0.016
VRSA	22524	ND	<0.078	200	0.016 - 0.031
VRSA	22525	ND	<0.078	100	0.016 - 0.031
VRSA	22526	ND	<0.078	<1.56	0.0156 - 0.031

VRSA	22527	ND	<0.078	1.56 - 3.13	0.031
VRSA	22528	ND	<0.078	>200	0.0156 - 0.031
VISA	21143	ND	<0.078	12.5	0.0156 - 0.031
VISA	21156	ND	>10	3.13 - 6.25	0.063
VISA	21157	ND	<0.078	12.5	0.031 - 0.063
VISA	21161	ND	<0.078	6.25	0.0156 - 0.031
VISA	21171	ND	>10	3.125	0.0156 - 0.031
VISA	21178	ND	<0.078	6.25	0.0156 - 0.031
VISA	21186	ND	>10	6.25	0.031
VISA	21352	ND	>10	6.25	0.0156 - 0.031
MRSE	925	0.062	ND	0.5	0.004
MRSE	928	0.5	ND	1.0	0.004
MRSE	927	0.125	ND	1.0	0.002
MRSE	926	0.062	ND	1.0	0.002
VRE	3322	>4	ND	0.25	0.016
VRE	3091	>4	ND	>4	0.002
VRE	3090	>4	ND	>4	>0.5
VRE	2967	>4	ND	>4	0.031
VRE	2966	>4	ND	>4	0.004
VRE	2138	>4	ND	>4	>0.5
VRE	2139	>4	ND	>4	0.002
VRE	2927	>4	ND	>4	0.008
B. anthracis	Ames	0.031	ND	ND	0.049
B. anthracis	Vollum V1B	0.125	ND	ND	0.098
C. difficile	9689	ND	ND	1.0	0.0078
C. difficile	43255	ND	ND	0.5	0.0630
C. difficile	43596	ND	ND	0.5	0.0310
C. difficile	43597	ND	ND	0.5	0.0310
C. difficile	BAA-1805	ND	ND	0.5	0.0310
C. difficile	BAA-1808	ND	ND	0.5	0.0310
C. difficile	BAA-1875	ND	ND	0.5	0.0156
C. difficile	BAA-1874	ND	ND	0.5	0.0078
C. difficile	700057	ND	ND	0.5	0.0078

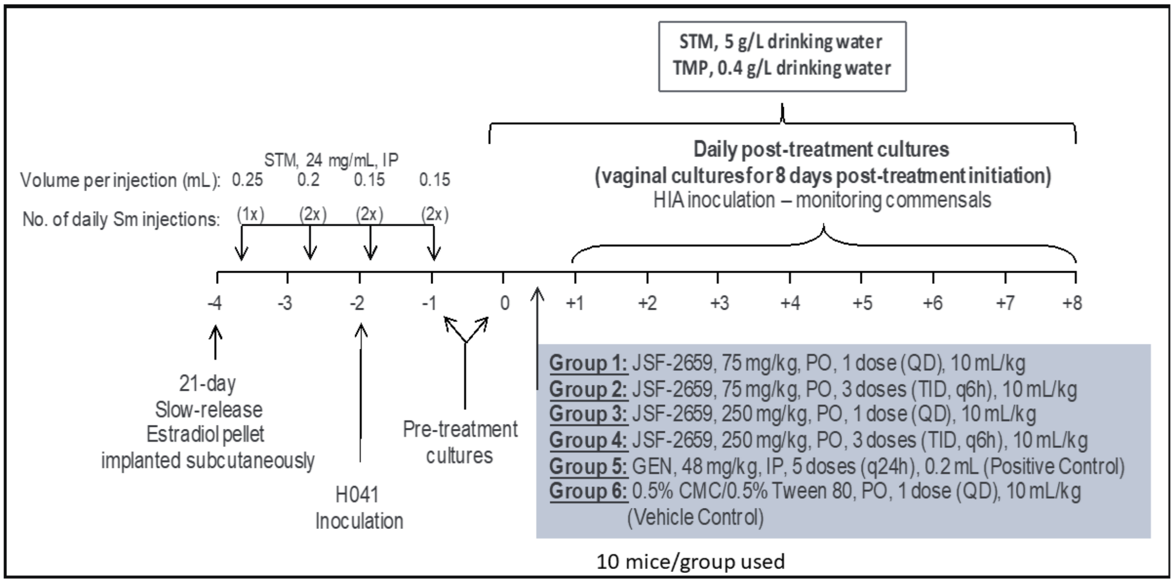

Suppl, Fig.1

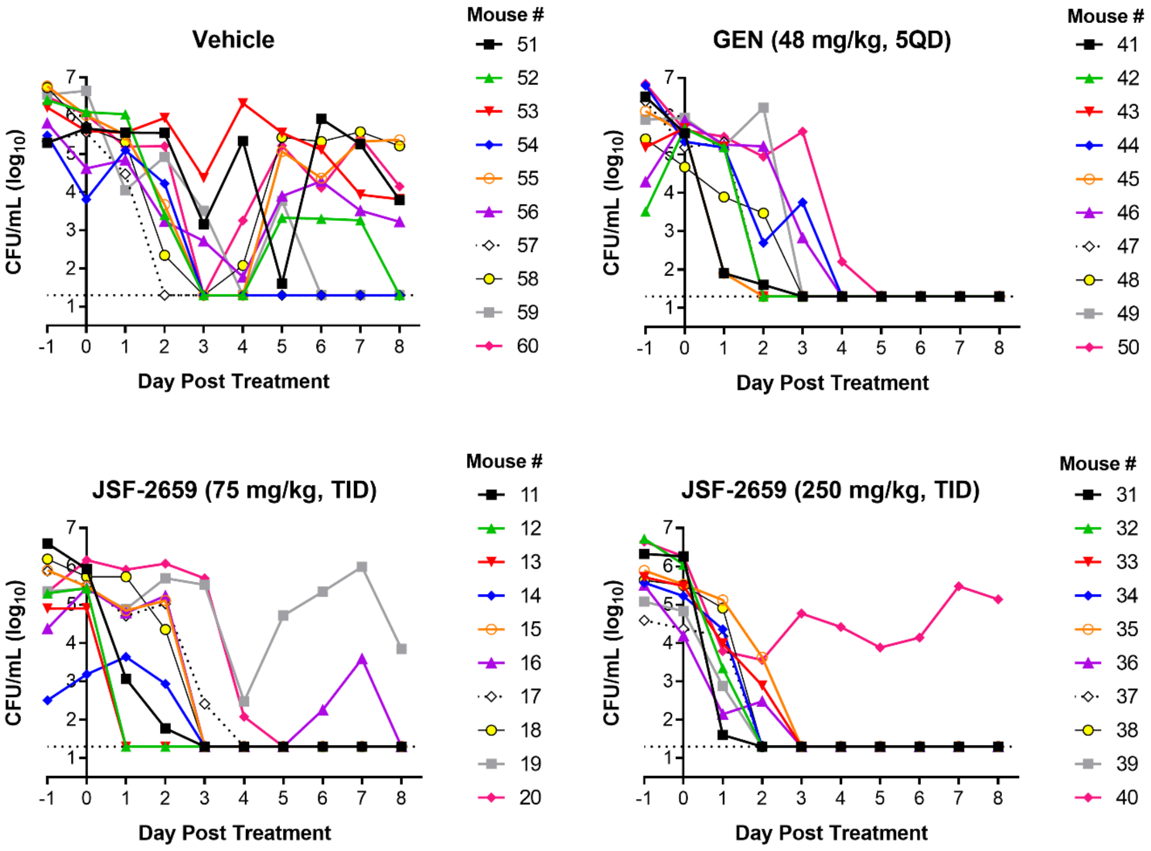

Suppl. Fig. 2
